# Impact of myelin phagocytosis on human myeloid cells and its effect on human oligodendrocytes

**DOI:** 10.1093/braincomms/fcag411

**Published:** 2026-11-03

**Authors:** Laura Eleonora Schmitz-Gielsdorf, Katharina Heß, Christian Thomas, Marie-France Dorion, Hannes Drexler, Stefanie Albrecht, Jack Antel, Inge Huitinga, Luisa H Klotz, Tanja Kuhlmann

**Affiliations:** Institute of Neuropathology, University Hospital Münster, Münster 48149, Germany; Institute of Neuropathology, University Hospital Münster, Münster 48149, Germany; Institute of Neuropathology, University Hospital Münster, Münster 48149, Germany; Neuroimmunology Unit, Montreal Neurological Institute, McGill University, Québec, Canada H3A 0G4; Max-Planck-Institute for Molecular Biomedicine, Mass Spectrometry Unit, Münster 48149, Germany; Institute of Neuropathology, University Hospital Münster, Münster 48149, Germany; Neuroimmunology Unit, Montreal Neurological Institute, McGill University, Québec, Canada H3A 0G4; Department of Neuroimmunology, Netherlands Institute for Neuroscience, Royal Netherlands Academy of Arts and Sciences, Amsterdam 1105 BA, The Netherlands; Swammerdam Institute for Life Sciences, Center for Neuroscience, University of Amsterdam, Amsterdam 1090 GE, The Netherlands; Department of Neurology, University Hospital Muenster, Münster 48149, Germany; Institute of Neuropathology, University Hospital Münster, Münster 48149, Germany

**Keywords:** multiple sclerosis, remyelination, macrophages, microglia, cholesterol

## Abstract

Multiple sclerosis is characterized by immune-mediated myelin destruction, leading to oligodendrocyte loss and neuronal degeneration. While remyelination can mitigate myelin loss, this process often fails in the multiple sclerosis lesions. The underlying cellular and molecular mechanisms of remyelination failure in multiple sclerosis remain elusive. Results from experimental models suggested that myelin phagocytosis shifts myeloid cells from a pro- to a more anti-inflammatory phenotype, thus promoting myelin repair; however, in aged animals, myelin debris clearance and remyelination are impaired, at least partially due to an impaired cholesterol degradation. In this study, we investigated the link between remyelination, myelin debris clearance and lipid droplet formation and how myelin uptake influences the inflammatory phenotype of primary human phagocytes using multiple sclerosis tissue samples, primary human myeloid cells and human-induced pluripotent stem cell-derived oligodendrocytes. In multiple sclerosis tissue samples, no correlation was found between the formation of lipid droplets and remyelination. *In vitro*, macrophages from healthy individuals phagocytosing control myelin inhibited oligodendrocyte differentiation despite switching from a pro- to anti-inflammatory phenotype independent of lipid droplets. Macrophages from people with multiple sclerosis, autoimmune encephalitis or stroke phagocytosed myelin similar to macrophages from healthy individuals but did not upregulate cytokine secretion and had no effect on oligodendroglial differentiation. In contrast, macrophages phagocytosing multiple sclerosis myelin overcame this silenced phenotype and their supernatants impaired oligodendroglial differentiation. In summary, we examine here the formation, abundance and localization of perilipin positive lipid droplets in myeloid cells and their correlation with remyelination in multiple sclerosis lesions. Furthermore, our *in vitro* data suggest that myelin-phagocytosing myeloid cells impair oligodendroglial differentiation regardless of their inflammatory phenotype. Additionally, they demonstrate the importance of myelin alterations in driving inflammation as well as impaired oligodendroglial differentiation and highlight the presence of significant differences between multiple sclerosis and its animal models.

## Teaser

Schmitz-Gielsdorf *et al*. report that human macrophages phagocytosing myelin switch from a pro- to an anti-inflammatory phenotype, yet still impair oligodendroglial differentiation. Macrophages from multiple sclerosis patients remain largely silent when exposed to control myelin, but exposure to multiple sclerosis myelin overcomes this silenced state and inhibits oligodendroglia differentiation.

## Graphical Abstract

**Graphical Abstract:**
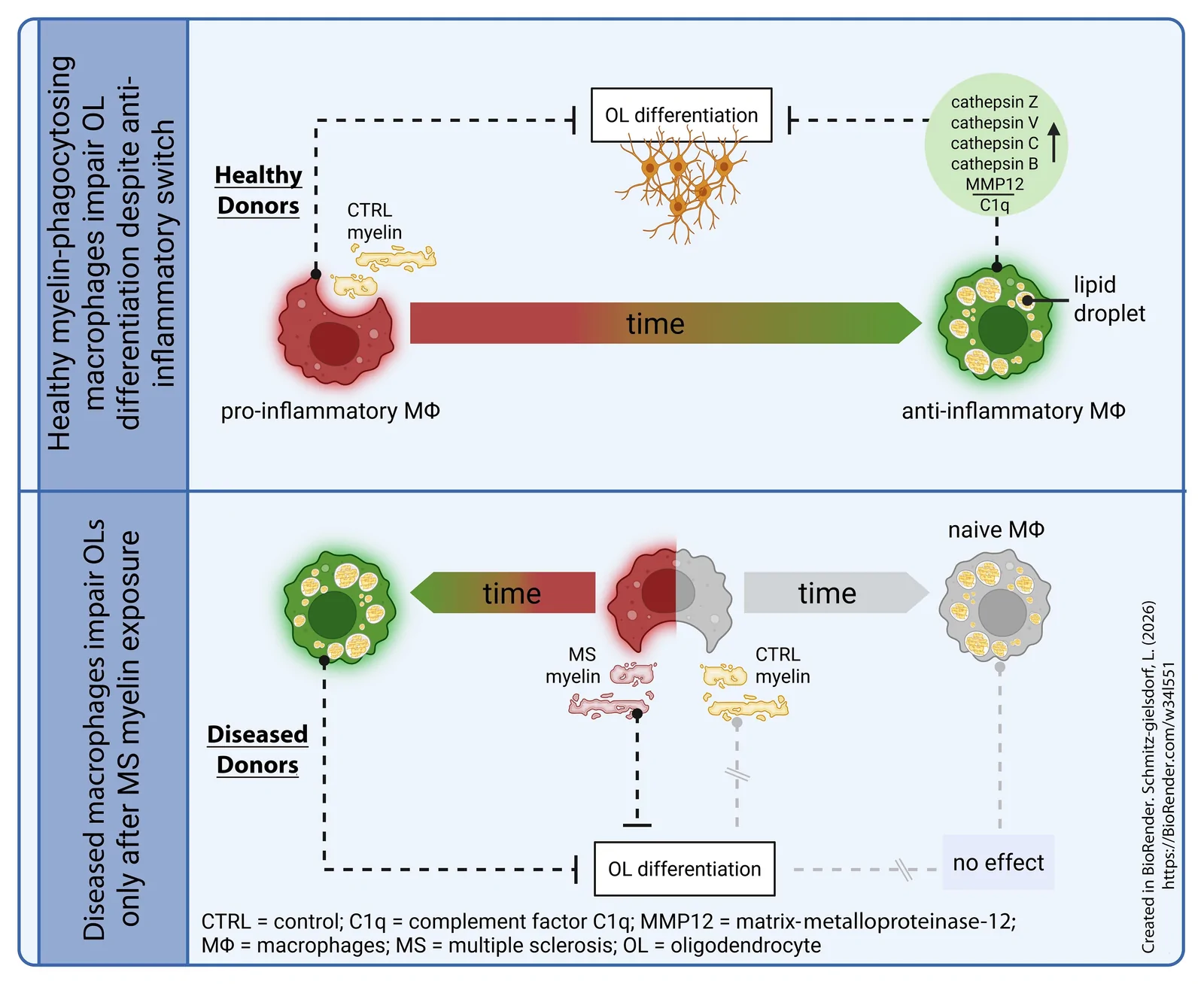

## Introduction

Multiple sclerosis is a chronic inflammatory disease of the CNS characterized by immune-mediated demyelination, oligodendrocyte loss, axonal degeneration, and neuronal damage. A hallmark of multiple sclerosis pathology is the abundant presence of blood-derived macrophages and brain-resident microglia (hereafter collectively referred to as myeloid cells) within active lesions and in rims of mixed active/inactive lesions.^1^ Although remyelination can partially restore lost myelin, this process frequently fails. Despite advancing immunotherapy, a treatment enhancing remyelination is still lacking, which highlights the need to understand the regenerative response of remyelination and the involvement of the immune system.^2^

Myeloid cells are often described along the M1/M2 paradigm, with pro- and anti-inflammatory properties respectively, although this classification is overly simplistic.^3^ While cells in the centre of active lesions express anti-inflammatory molecules, they lose their homeostatic signature, express M1- and downregulate M2-associated markers in the rim of mixed lesions and in active lesions during early stages of demyelination.^4-6^ Single-nuclei RNA-sequencing has identified a multiple sclerosis–specific subset, termed ‘microglia inflamed in multiple sclerosis,’ resembling disease-associated microglia observed in other neurodegenerative disorders.^7^ Myelin-laden myeloid cells in active and mixed lesions acquire an intermediate phenotype marked by concurrent expression of M1- and M2-associated markers.^6,8^ Remyelination is typically observed at lesion borders but is absent in mixed lesions with dense myeloid rims, suggesting that these cells may generate a non-permissive environment.^5^

*In vitro*, M1-conditioned supernatants impair the differentiation of human A2B5^+^ OPCs and induced pluripotent stem cell (iPSC)-derived oligodendrocytes (OLs), while M2-conditioned media seem to promote OL maturation in rodent *in vitro* studies.^5,9,10^ This beneficial effect, however, has not been confirmed in humans yet. In animal models, initiation of remyelination is associated with an M1-to-M2 shift, and depletion of macrophages during early repair phases disrupts oligodendrogenesis, indicating a supportive role for these cells.^10-14^

The impact of myelin uptake on myeloid phenotypes remains unclear. While some studies report an exacerbated M1 phenotype, others describe a reduction of the pro-inflammatory response or no effect.^15-19^ Phagocytosis of lipid-rich structures, such as myelin, induces lipid droplet (LD) accumulation in myeloid cells. In demyelinating animal models, lipolysis-mediated LD degradation enhanced remyelination, while defective cholesterol clearance impaired repair.^20,21^ Adequate sterol metabolism within myeloid cells appears critical for both remyelination and resolution of inflammation.^22^ Conversely, impaired LD biogenesis has also been linked to inefficient regeneration, suggesting divergent roles of lipid handling.^23^ Human data on this matter are still lacking.

In this study, we examined the correlation between remyelination and cholesterol degradation in multiple sclerosis tissue samples and investigated how myelin uptake modulates the inflammatory phenotype of primary human macrophages and microglia, before and after LD formation, and their subsequent impact on human iPSC-derived OLs. We found no association between LD accumulation and remyelination, indicating that cholesterol metabolism defects are not a dominant cause of failed repair. Instead, *in vitro* myelin-laden macrophages inhibited OL differentiation despite transitioning from a pro- to anti-inflammatory phenotype, and this inhibitory effect was independent of LD formation. Moreover, multiple sclerosis–derived macrophage as well as macrophages derived from individuals with stroke or autoimmune encephalitis displayed reduced responses to control myelin. Interestingly, the negative effect of macrophages on oligodendroglial differentiation was restored upon uptake of multiple sclerosis myelin, suggesting that disease-related alterations of myelin itself contribute to impaired repair in multiple sclerosis.

## Material and methods

### Human tissue samples and cells

The study was performed retrospectively on a collection of 29 paraffin-embedded brain biopsies from 29 patients. None of the study authors were involved in decision making with respect to biopsy. Furthermore, human iPSC-derived oligodendrocytes and PBMCs were included into the study, details are provided below. The study was approved by the Ethics Committee of the University of Münster (Az: 2012-407-f-S, Az: 2017-360-f-S, 2016-026-f-S).

### Histological classification of multiple sclerosis lesions

All lesions met the established histological criteria for an inflammatory demyelinating lesions, and were categorized either as active and demyelinating lesions (*n* = 25) or active and post-demyelinating lesions (*n* = 5) based on the updated histological classification of multiple sclerosis lesions by Kuhlmann *et al*.^1^ Demyelination was identified through immunohistochemistry (IHC) for MBP, while the number and distribution of myeloid cells—specifically, infiltrating blood-derived monocytes and CNS-resident microglia—were determined via CD68 staining. Active lesions display a high numbers and densities of CD68^+^ macrophages/microglia throughout the entire lesion compared to the directly adjacent periplaque white matter or normal-appearing white matter (NAWM). Phagocytes within active and post-demyelinating lesions lacked myelin breakdown products, in contrast to active and demyelinating lesions where phagocytes contained myelin degradation products such as MBP, PLP, LFB, CNP or MOG.

### Immunohistochemistry

IHC was performed on paraffin-embedded 4-µm tissue sections using the Dako REAL Detection System (#DC137C100), Dako) and an automated immunostainer (AutostainerLink 48, Dako). After heat-induced epitope retrieval treatment and peroxidase block (#S2023, Dako), sections were incubated with primary antibodies, followed by biotinylated secondary antibodies (#PD000RP, DCS) and streptavidin/peroxidase (#PD000RP, DCS). Diaminobenzidine (#DC137C100, DCS) served as chromogen, nuclei were counterstained with haematoxylin (#S3301, Dako).

Primary antibodies: MBP (1:200, #ab7439, Abcam), CD68 (1:200; #M0814, Dako) and NogoA (1:20.000; NogoA) were a generous gift from M.E. Schwab, Brain Research Institute, University of Zürich and Department of Biology, Swiss Federal Institute of Technology Zürich, Switzerland), BCAS (1:500, #sc-393740, SantaCruz), OLIG2 (1:150; #18953, IBL) and PLIN2 (1:150; #GP40, Progen). Positive cells were quantified at 40-fold magnification using a morphometric grid, and counts per square millimetre were calculated.

To sub-categorized the lesions with respect to the presence or absence of MBP-containing and PLIN2^+^ myeloid cells, tissue sections were stained for MBP, CD68 and PLIN2. Active lesions containing MBP^+^ myeloid cells were categorized as MBP^+^. The number of CD68^+^ and PLIN2^+^ myeloid cells was quantified in consecutive tissue sections, and the proportion of PLIN2^+^ cells relative to CD68^+^ cells per lesion was determined (range 0% to 69%, median = 9.4%, mean ± SEM = 24.8 ± 4.7%). Lesions in which the proportion of PLIN2^+^ cells was less than 11% of the total CD68^+^ myeloid cells were classified as PLIN2^−^ (*n* = 16; range 0% to 10.9%, median = 2.6, mean ± SEM = 3.6 ± 0.9%), whereas lesions with more than 11% were classified as PLIN2^+^ (*n* = 13; range 24.7% to 68.7%; median = 50.4%, mean ± SEM = 51.3 ± 3.2%).

Remyelination was assessed using a semiquantitative score as in previous studies.^5^ In brief, in biopsies, remyelination was identified as thin, irregular formed myelin sheaths utilizing IHC for MBP. Because biopsy samples frequently display only parts of the lesion, we quantified the extent of remyelination using the following categories as arbitrary score: 0 = complete absence of remyelination, 1 = individual oligodendrocytes extending remyelinating processes, 2 = patchy remyelination, 3 = remyelination throughout the sampled lesion area (Supplementary Fig. 1).

### Myelin isolation

Murine myelin was isolated from brain and spinal cord from 8–9-week-old C57BL/6 mice, independent of their sex. The animals used in this study were provided by the Zentrale Tierexperimentelle Einrichtung, University Hospital Münster, Germany. All experimental procedures were approved by the ‘Landesamt für Natur, Umwelt und Verbraucherschutz Nordrhein-Westfalen’ (LANUV; AZ: 81-02.05.50.17.017, 81-02.05.50.22.019).

Isolation from frozen tissue followed Norton and Poduslo.^24^ Tissue was sonicated in 0.35 M sucrose, layered onto 0.85 M sucrose and centrifuged at 107,000×g for 30 min at 4°C without acceleration and break. The interphase was collected, washed 4 × in ice-cold ddH20 to remove debris, and the gradient centrifugation/washing repeated. Purified myelin was resuspended in DPBS (D8537-1L Sigma), stored at −80°C, and quantified via Bradford assay (#500-0205, Biorad). Human control and multiple sclerosis myelin were kindly provided by the Netherland Brain Bank and isolated as described previously.^24,25^ The multiple sclerosis NAWM myelin samples were derived from five independent multiple sclerosis patients, each representing a separate preparation. Control NAWM myelin was obtained from two independent individuals presenting with mild or frontal atrophy; although no formal diagnosis was reported, dementia was suspected. Each control sample also represented an independent preparation. Due to limited material availability and to reduce inter-sample variability, multiple sclerosis myelin samples were pooled, as were control myelin samples, prior to downstream analyses. A heatmap comparing the abundance of key myelin peptides from multiple sclerosis and control myelin is displayed as Supplementary Fig. 2.

### Isolation of human PBMCs

PBMCs were isolated from buffy coats by density gradient centrifugation. Diluted blood was layered onto Lymphopure (#426202, Biolegend) and centrifuged at 800×g for 20 min without acceleration and break. The interphase was washed twice in DPBS at 300×g for 10 min. Aliquots of 1 × 10^7^ PBMCs were frozen in RPMI (#21875034, Thermo) + 10% DMSO (#D2650, Sigma) and stores in liquid nitrogen. Patient cells were provided by the UKM Neurology Biobank (20 patients with 55% female, Supplementary Table 1); healthy controls from UKM thrombocyte donation services.

### Differentiation of human blood–derived macrophages

Thawn PBMCs were seeded in RPMI + 10% Penicillin/Streptomycin (Pen/Strep; #P4333, Sigma) onto uncoated multiwell cell culture plates (2.5 × 10^7^ cells/24 well; 5 × 10^7^ cells/12 well; 1 × 10^7^ cells/6 well). After 4 h, non-adherent cells were washed off and cultures maintained in Blood-derived macrophage medium (BMM: RPMI + 5% FBS (#F7524, Sigma) + 1% Pen/Strep) overnight. Medium was replaced with BMM + 50 ng/ml M-CSF (#300-25, Peprotech) and renewed every 3 days. On day 7, the cells were either polarized or treated with 12.5 µg myelin/250.000 cells for respective timepoints. M1 polarization: 50 ng/ml IFNγ (1 h; #506532, Biolegend) followed by 10 ng/ml LPS (18 h, #L2603, Sigma). M2 polarization: 20 ng/ml IL4 (72 h; #200-04, Peprotech). Untreated cells served as M0.

### Human NPC culture

NPC were cultured according to Reinhard *et al*.^26^ Shortly, NPC were cultured in N2B27 medium (1:1 premixed neurobasal medium (#21103-049, Gibco) and DMEM/F12 (#21331-020, Gibco), 1% B27 supplement without vitamin A (#12587-010, Gibco), 0.5% N2 supplement (#17502-048, Gibco), 1% Pen/Strep, 2 mM L-glutamine (#G7513, Sigma)) supplemented with 1 μM SAG (#11914, Cayman Chemical Company), 3 μM CHIR99021 (#1386, AxonMedChem) and 200 μM L-ascorbic acid (#A4544, Sigma). At 80% confluency, cells were dissociated with pre-warmed accutase (#A6964, Sigma), centrifuged at 200×g for 5 min, resuspended and seeded onto Geltrex (#A1413302, Gibco) or Cultrex (#3536-005-02, R&D) coated well-formats. Medium was changed every second day.

### Differentiation of human-induced oligodendrocytes

Human-induced oligodendrocytes were differentiated from NPC lines with a stably integrated doxycycline-inducible SON (SOX10, OLIG2, NKX6.2) cassette, following the published protocol.^27,28^ We used three lines, originating from one male and two female donors. NPCs were seeded (100.000 cells/12 well) in NPC medium (supplemented N2B27, see above) on Geltrex or Cultrex. 1 µg/ml doxycycline (#D9891, Sigma) was added the next day (day −2). On day 0, the medium was changed to glial induction medium (GIM: DMEM/F12, 1% B27 supplement without vitamin A, 0.5% N2 supplement, 1% Pen/Strep, 2 mM L-glutamine, 1 μM SAG, 200 μM L-ascorbic acid, 10 ng/ml T3 (#T6397, Sigma), 10 ng/ml IGF-1 (#100-11, Pepro Tech), 10 ng/μl PDGF-AA (#100-13A, Pepro-Tech), 10 ng/μl human NT3 (#450-03, Pepro-Tech), 1:1000 Trace element B (#25022CI, Corning) and 1 µg/ml doxycycline. On day 2, medium was changed to glial differentiation medium (GDM: DMEM/F12, 1% B27 supplement without vitamin A, 0.5% N2 supplement, 1% Pen/Strep, 2 mM L-glutamine, 200 μM L-ascorbic acid, 60 ng/ml T3, 10 ng/ml IGF-1, 10 ng/μl human NT3, 200 μM dbcAMP (#D0627, Sigma), 1:1000 Trace element B and 1 µg/ml doxycycline. On day 7, cells were replated on poly-L-lysine (PLL; #P4707, Sigma)/laminin (#L2020, Sigma). Doxycycline was withdrawn at day 16. Medium was changed every other day.

### Primary human microglia

Microglia RNA and supernatants were kindly provided by the group of Jack Antel (Montreal Neurological Institute, Canada). Human brain tissues were obtained with consent and approval from the local ethics boards at the Montreal Neurological Institute and the Montreal Children's from epilepsy surgeries, distant from epileptic foci. Glial cells were isolated as previously described using mechanical/chemical digestion, and Percoll (Sigma-Aldrich) gradients, seeded overnight and purified by adhesion.^29^ Cultures were maintained in Minimum Essential Medium (MEM; Sigma Aldrich) supplemented with 1% GlutaMAX (Thermo Fisher Scientific), 1% Pen/Strep, 0.1% glucose, and 5% FBS. Microglia were left untreated or treated with 12.5 µg myelin/250,000 cells for respective timepoints.

### Immunocytochemistry

For immunocytochemistry (ICC) cells were fixed with 4% PFA (#200-001-8, Merck) for 20 min at RT and washed thrice. Cells were permeabilized with 0,05% TritonX (skipped for O4) for 10 min, blocked in DPBS with 5% FBS/NGS (G6767, Sigma) for 1 h at RT. Primary antibodies in blocking solution were applied overnight at 4°C. The next day cells were incubated with respective secondary antibodies for 1 h at RT. Nuclei of cells on plastic cell culture plates were counter stained with DAPI for 10 min at RT and analysed with a Leica DMI1600B inverted microscope. Cells were washed thrice before and after incubation with DAPI (#D9542, Sigma). Cells on glass coverslips were washed thrice with DPBS and once with ddH2O before being mounted in Roti Mount Fluor Care DAPI (#HP20.1, Roth). Images of cover slips were acquired on a Zeiss LSM700 confocal microscope. Applied antibodies were the following: ms α-O4 (1:100; #MAB1326, R&D); rt α-MBP (1:4000; #ab7349, Abcam); ms α-CD68 (1:200; #M0814, Dako); rb α-PLIN2 (1:50; #HPA016607, Merck); rb α-iNOS (1:400; #06-573, Millipore); rb α-CD206 (1:500; #HPA004114, Sigma); AlexaFluor488 Anti-Rabbit (1:250; #111-545-144, Jackson/Dianova); Cy3 Anti-Mouse (1:250; 115-165-062, Jackson/Dianova); AlexaFluor488 Anti-Rat (1:250; 112-545-167); AlexaFluor647 Anit-Rat (1:250; #2119156, Thermo); Cy3 Anti-rabbit (1:250; #111-165-144, Jackson/Dianova).

### RNA isolation

RNA was isolated using the GenElute Mammalian Total RNA Miniprep Kit (#RTN350-1KT, Sigma). After removal of unphagocytosed myelin, cells were lysed in lysis buffer containing β-mercaptoethanol. Equal volume of 70% Ethanol was added to lysate and transferred to a nucleic acid binding column. Flow through was discarded and the column washed with Wash Solution 1. Samples were incubated for 15 min at RT with DNase, followed by washing steps with Wash Solution 1 and 2. RNA was eluted with 50 µl Elution Solution and stored at −80°C.

### RNA sequencing

RNA-sequencing data from macrophages of three independent donors were analysed. Transcript-level expression was quantified directly from raw sequencing reads (.fastq format) using Salmon (v1.10). All downstream analyses were performed in R (v4.3.0). Gene-level count summarization and integration across samples were conducted with tximport (v1.28), after which the resulting tximport object was converted into a DESeqDataSet using DESeq2 (v1.40.1). Genes with at least 10 counts were considered expressed. Raw counts were transformed using the variance stabilizing transformation (VST) implemented in DESeq2 for visualization and clustering. Normalized count data are provided in the Supplementary material.

To characterize transcriptomic polarization signatures of polarized and myelin-phagocytosing macrophages, we performed differential gene expression analyses using DESeq2. Specifically, untreated M0 macrophages were compared to polarized (M1/M2) or myelin-phagocytosing macrophages (mye(6 h)/mye(24 h)), and mye(24 h) macrophages were compared to M2. For functional interpretation, enrichment analyses were performed using EnrichR with the Reactome2022 database, and data visualization was carried out in GraphPad Prism.

### ELISAs

Cytokine production was quantified by ELISA Kits for IL10 (#29-8109-65, Thermo); IL1β (#88-7261-86, Thermo); TNFα (#29-8329-64, Thermo) and IFNγ (#88-7316-76, Thermo) according to manufacturer’s instructions. Plates (Corning Costar 9018) were coated at 4°C overnight with capture antibody (1:250 in coating buffer), washed and blocked with ELISA/ELISPOT for 1 h at RT. Standards were reconstituted in ELISA/ELISPOT for 30 min, diluted and applied with samples for overnight incubation at 4°C. After washing, detection antibodies were added for 1 h at RT, followed by Streptavidin-HRP or Avidin-HRP for 30 min. Plates were washed, developed with TMB for 15 min, stopped with Stop Solution (#SS04, Thermo) and absorbance measured at 450 nm.

### CellTiterGlo

Viability of cells was determined using the CellTiter-Glo 2.0 Cell Viability Assay (#G9242, Promega). Reagent was thawed at 4°C overnight, equilibrated with culture plates to 22°C for 30 min and added in equal medium. After 2 min lysis on an orbital shaker and 10 min stabilization, suspensions were transferred to opaque plates and luminescence recorded.

### Mass spectrometry

Proteins from the myelin preparations were first delipidated by acetone precipitation in the presence of 20 mM NaCl, air-dried, and resuspended in 50 µl RIPA buffer for sample preparation using the protein aggregation capture protocol^30^ with slight modifications. Briefly, solubilized proteins were subjected to reductive alkylation by the addition of 5 mM tris(2-carboxyethyl) phosphine and 10 mM chloroacetamide, followed by heating at 95 °C for 10 min. After cooling to room temperature, acetonitrile was added to a final concentration of 70%, and 250 µg of carboxy-activated magnetic beads (PureCube Carboxy MagBeads, #50201; Cube Biotech) were added to induce protein aggregation on the bead surface for 20 min. The supernatant was removed using a magnetic stand, and beads were washed once with 100% acetonitrile and once with 70% ethanol. Beads were resuspended in 50 mM Tris-HCl, pH 8.5, and proteins were digested with LysC (1 µg) for 1.5 h at 37 °C on a thermomixer (1000 rpm), followed by the addition of trypsin (1 µg) and overnight incubation under the same conditions. Digestion was stopped by the addition of trifluoroacetic acid to a final concentration of 1%. Resulting peptides were desalted using SDB-RPS tips (Affinisep). To ensure complete removal of bead material, desalted peptides were further purified using bead removal filters (BioSPE BeadRem, Affinisep). Finally, peptides were resuspended in 15 µl of Buffer A (0.1% formic acid in water), and peptide concentrations were estimated by measuring absorbance at 280 nm.

Nanoflow reversed-phase liquid chromatography was performed on a nanoElute 2 UHPLC system coupled online to a timsTOF Pro2 mass spectrometer via a CaptiveSpray nano-electrospray source (all from Bruker Daltonics). For peptide separation, a 25 cm × 75 µm fused-silica emitter capillary packed with 1.9 µm ReproSil-Pur 120 C18 resin (Dr. Maisch; capillary and packing by ProtumLink, Jena) was used. Peptides were separated with a linear gradient increasing from 4% to 27% Buffer B (0.1% formic acid in acetonitrile) over 60 min at a flow rate of 300 nL/min, followed by a step to 95% Buffer B for 5 min and subsequent re-equilibration to starting conditions. The mass spectrometer was operated in DIA-PASEF mode using the standard long-gradient dia-PASEF method. Spectra were recorded across a mass range of 100–1700 m/z and an ion mobility range of 1/K_0_ = 0.60–1.60 Vs/cm^2^, with a TIMS ramp time of 100 ms. Collision energy was dynamically adjusted according to ion mobility, decreasing from 59 eV at 1/K_0_ = 1.60 Vs/cm^2^ to 20 eV at 1/K_0_ = 0.60 Vs/cm^2^.

Raw mass spectrometry data were processed separately for human and mouse samples using DIA-NN (v2.0.2) in library-free mode with heuristic protein inference at the gene level. For human datasets, match-between-runs (MBR) was enabled. Quantification was performed using QuantUMS (Quantification Using an Uncertainty Minimizing Solution). UniProt reference proteomes used were UP000000589_1009.fasta (mouse) and UP000005640_9606.fasta (human), both in versions from April 2024 without additional isoform sequences.

Search parameters included as fixed modification carbamidomethylation on cysteine; variable modifications: methionine oxidation, protein N-terminal acetylation and citrullination of arginine residues (mass shift: 0.984016 Da; UniMod:1). Trypsin was set as the digesting protease, allowing up to one missed cleavage. Peptide length was restricted to 7–30 amino acids. Mass tolerances were set to 20 ppm for precursor (MS1) and 15 ppm for fragment ions (MS2). Charge states from +1 to +5 were considered. False discovery rate (FDR) thresholds were applied at 1% at both peptide and protein levels. Only proteins expressed ≥2-fold higher in the multiple sclerosis sample were selected for further analysis, and afterwards filtered for immune- or multiple sclerosis-associated proteins.

### Experimental design and statistical analysis

Experiments with primary cells included technical replicates and were repeated at least three times with biological independent samples. Images from all replicated were acquired and quantified blinded. Statistical analysed were performed in GraphPad Prism 8 (GraphPad Software Inc., San Diego, CA, USA). Results are presented as means ± SEM. Multiple group comparisons used mixed-effects analysis or Tukey-corrected one-way ANOVA. *P* values <0.05 were considered significant (*P < 0.05, **P < 0.01, ***P < 0.001).

## Results

### Lipid droplet formation shows no correlation to oligodendrocyte number and remyelination in human multiple sclerosis lesions

Since in demyelinating animal models, the formation and persistence of PLIN2^+^ LDs has been associated with impaired remyelination,^20^ we examined its relationship to oligodendrocyte numbers and remyelination in human active multiple sclerosis lesions. Lesions were sub-categorized by presence or absence of MBP-containing and PLIN2^+^ myeloid cells.

No differences were observed in numbers of NogoA^+^, OLIG2^+^ or BCAS^+^ oligodendrocytes or remyelination scores between active lesions with or without PLIN2^+^ myeloid cells (Fig. 1A–E), suggesting that formation of PLIN2^+^ LD following myelin uptake by myeloid cells does not impact the presence of oligodendrocytes or remyelination.

**Figure 1:**
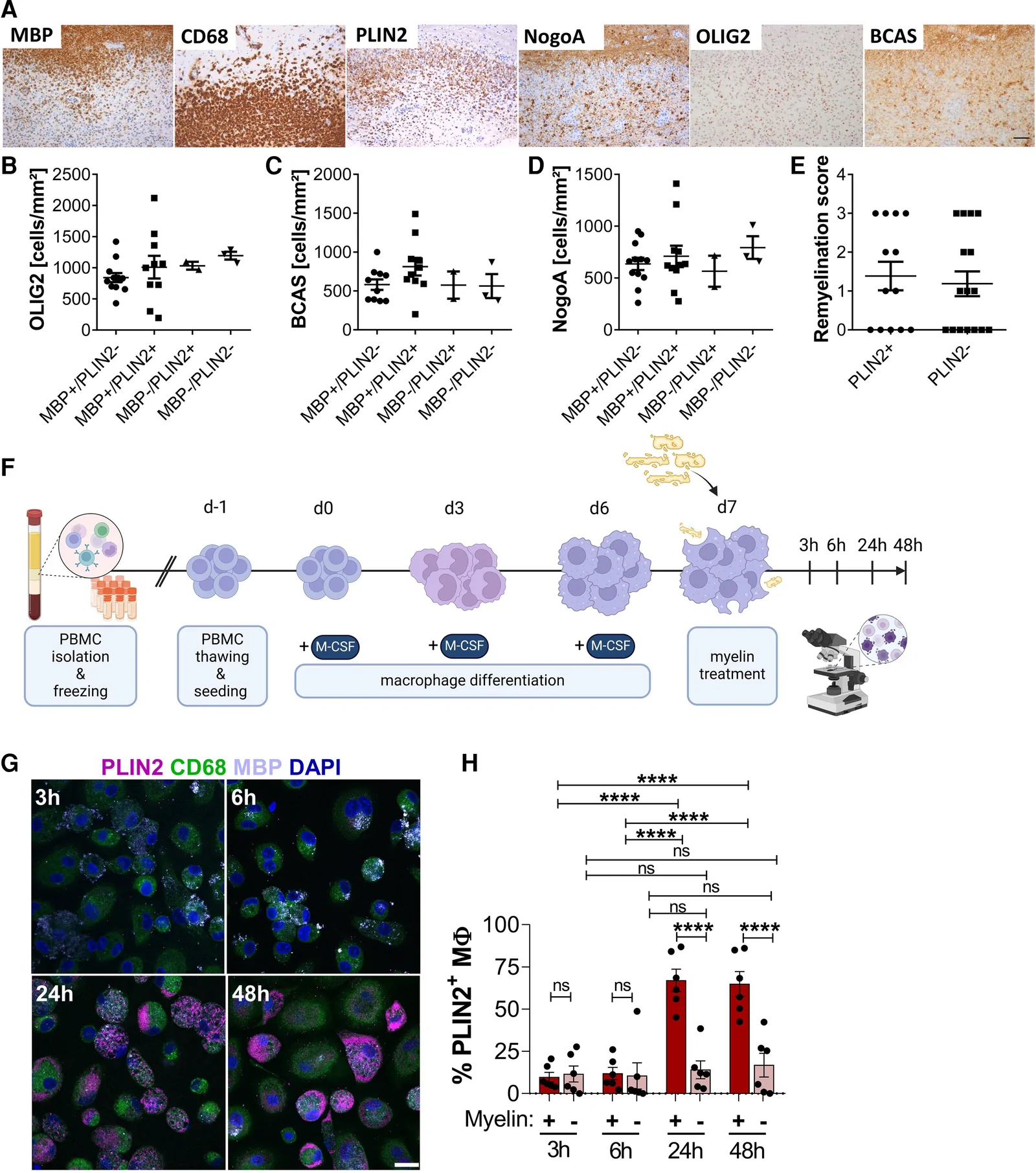
**Lipid droplet formation shows no correlation to oligodendrocyte number and remyelination in human multiple sclerosis-lesions and myelin uptake induces lipid droplet formation after 24 h in healthy blood-derived macrophages *in vitro*. (A)** Immunohistochemistry (IHC) of active demyelinating multiple sclerosis lesions for myelin marker myelin-basic protein (MBP), pan-macrophage marker CD68, lipid droplet marker perilipin 2 (PLIN2), oligodendrocyte markers NogoA, OLIG2 and BCAS. **(B)** Quantification of OLIG2 (F (DFn, DFd): F (3, 23) = 0.7566), **(C)** BCAS (F (DFn, DFd): F (3, 21) = 1.309) and **(D)** NogoA (F (DFn, DFd): F (3, 24) = 0.4654) showed no significant differences between lesions with the presence or absence of MBP containing and/or PLIN2^+^ macrophages. **(E)** Remyelination score is independent of the absence or presence of PLIN2^+^ lipid droplets. *N* = 13 PLIN2^+^ lesions, 16 PLIN2^−^ lesions. Each point represents a lesion. *P* = 0.6875 **(F)** Schematic of experimental design. Created in BioRender. Schmitz-Gielsdorf, L. (2026) https://BioRender.com/jbvds8i  **(G)** ICC of blood-derived macrophages for pan-macrophage marker CD68, lipid droplet marker PLIN2, myelin marker MBP and nuclear marker DAPI at different timepoints of myelin treatment (3, 6, 24 and 48 h). **(H)** Quantification of respective ICC shows a significant increase in lipid droplet formation 24 and 48 h after uptake of myelin (F (DFn, DFd): F (2.052, 10.26) = 23.47). Red: with myelin, light pink: without myelin. *N* = 6 as biological replicates from three independent macrophage differentiations out of two independent PBMC isolations. All comparisons are indicated in the graph; mean ± SEM (*P < 0.05, **P < 0.01; ***P < 0.001; ****P < 0.0001; one-way ANOVA with Tukey’s, DFd, degrees of freedom denominator; DFn, degrees of freedom numerator; F, f-value. Scale bars in A = 50 µm and in G = 20 µm.

### Myelin uptake induces a switch from an initially pro- to an anti-inflammatory phenotype, correlating with LD formation in blood-derived macrophages

To examine how myelin-phagocytosis affects macrophages and oligodendrocytes at distinct phases of myelin processing, we treated human peripheral blood mononuclear cell (PBMC)-derived macrophages with mouse brain myelin, due to high similarity to human myelin.^30^ PBMCs from healthy human individuals were differentiated into mature macrophages, treated with myelin for defined intervals and assessed for lipid droplet formation and myelin phagocytosis after 6, 12, 24 and 48 h (Fig. 1F). ICC for PLIN2 revealed significant LD formation at 24 and 48 h after myelin treatment (67.00% ± 6.635 and 64.85% ± 7.375) versus untreated controls (14.06% ± 5.254 and 16.78% ± 7.035) (Fig. 1G and H). MBP immunostaining showed myelin uptake as early as 3 h and to a higher degree after 6 h. Hence, we defined the ‘initial uptake’ to be after 6 h and the ‘LD forming’-phase after 24 h of myelin treatment. For simplicity these macrophages will be referred to as mye(6 h)-macrophages or mye(24 h)-macrophages.

To dissect how myelin uptake affects the inflammatory phenotype of blood-derived macrophages, we analysed their transcriptome and secretome and compared them with unpolarized (M0) and classical M1 or M2 polarized macrophages by assessing transcriptomic changes and cytokine secretion using bulk RNA sequencing and ELISA for interleukin 10 (IL10), interferon γ (IFNγ), interleukin 1β (IL1β) and tumor necrosis factor α (TNFα) (Figs 2 and 3, Supplementary Table 4).

**Figure 2:**
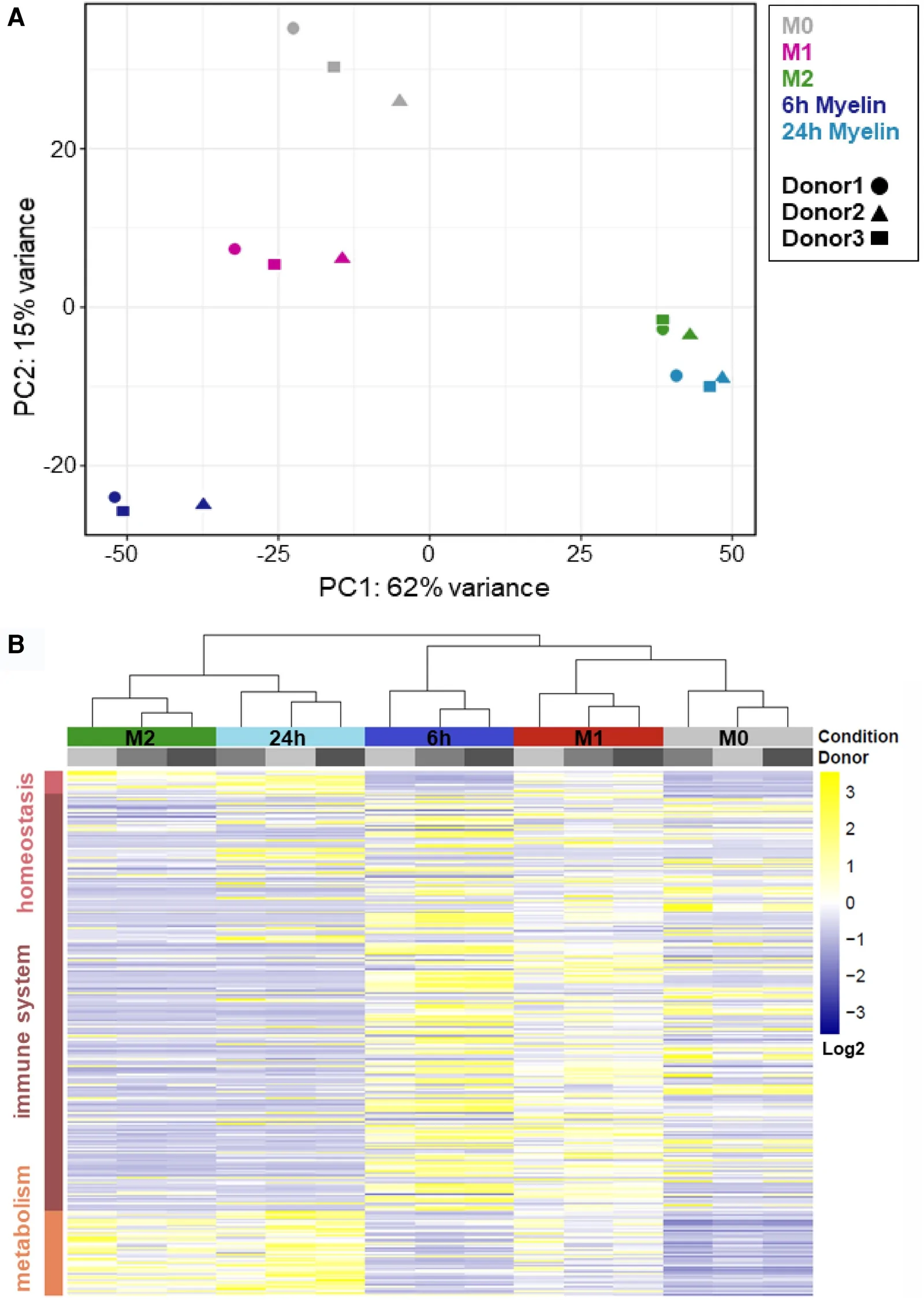
**Transcriptomic similarities between myelin-treated and M2-like macrophages. (A)** Principal component analysis (PCA) confirms that mye(24 h)-macrophages cluster closely together with anti-inflammatory (M2)-like macrophages. (**B**) Analysis of up- and downregulated genes showing that mye(24 h)-macrophages and anti-inflammatory (M2)-like macrophages display a similar expression pattern. *N* = 3 macrophage generations from 3 donors.

**Figure 3:**
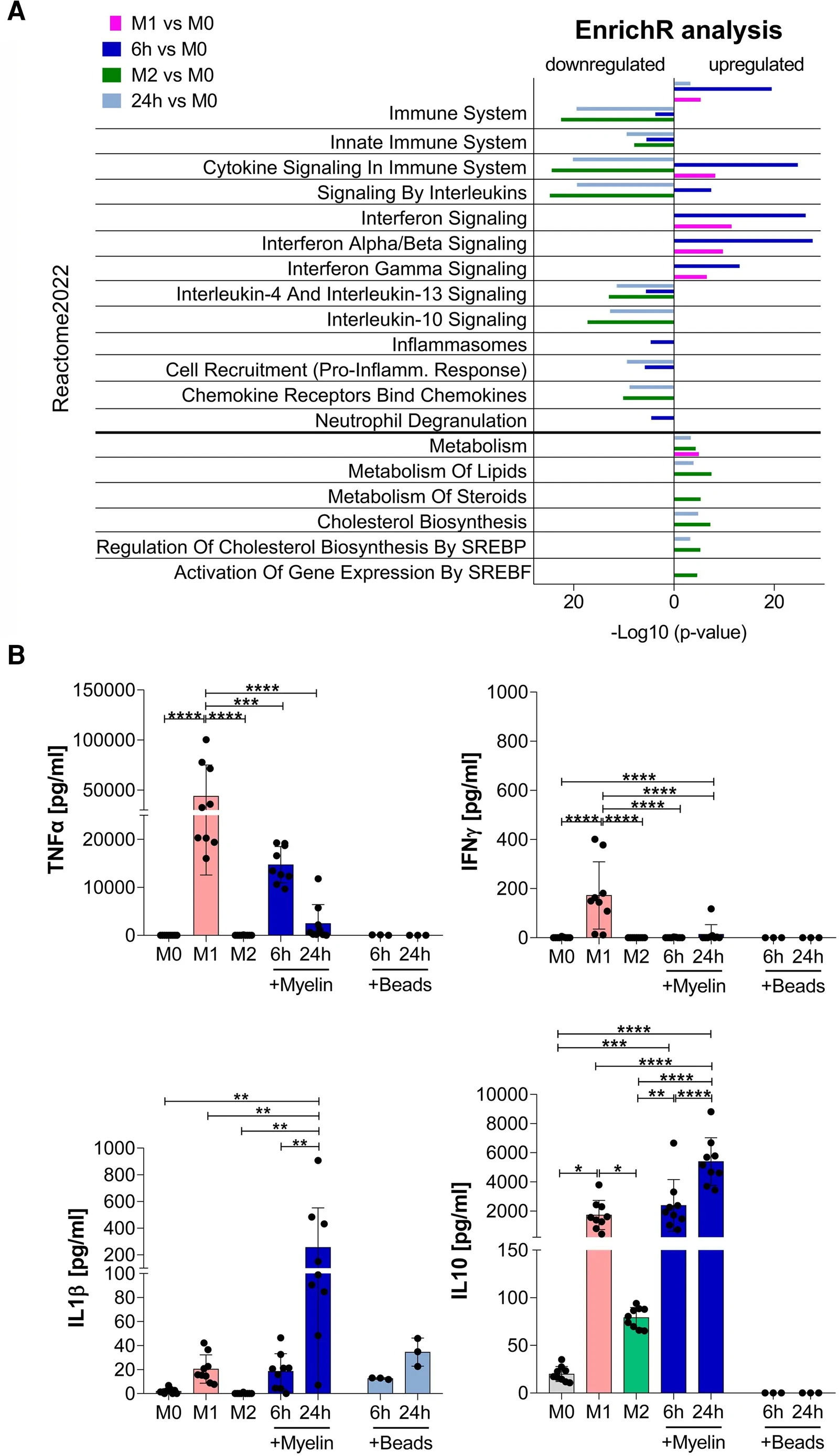
**Phenotypic switch from pro-inflammatory to anti-inflammatory after prolonged myelin-uptake in healthy blood-derived macrophages.** (**A**) EnrichR pathway analysis of up- and downregulated genes shows similarities between mye(6 h)-macrophages and pro-inflammatory M1 and mye(24 h)-macrophages and anti-inflammatory M2. Transcriptome analysis was performed in one independent experiment with three biological replicates, *n* = 3. (**B**) Analysis of mye(6 h)- and mye(24 h)-macrophage conditioned medium via ELISAs for TNFα (F (DFn, DFd): F (6, 44) = 12.19), IL1β (F (DFn, DFd): F (6, 44) = 4.610), IFNγ (F (DFn, DFd): F (6, 44) = 9.602) and IL10 (F (DFn, DFd): F (6, 44) = 25.89). Secretome analysis was performed in five independent macrophage generations, with a total of nine donor cells as biological replicates. Mean ± SEM (*P < 0.05, **P < 0.01; ***P < 0.001; ****P < 0.0001; one-way ANOVA with Tukey’s,). Each condition was compared to each other condition; only significant differences are depicted in the graphs. DFd, degrees of freedom denominator; DFn, degrees of freedom numerator; F, f-value.

Principal component analyses (PCAs) revealed distinct M0, M1 and M2 macrophages clusters; mye(24 h) clustered with M2, while mye(6 h) formed a separate cluster (Fig. 2A and B). EnrichR analysis (Database Reactome2022) of up- and downregulated genes in myelin-treated or M1/M2 polarized macrophages in comparison to untreated M0 macrophages revealed that differentially expressed genes were mostly involved in pathways, either related to ‘Immune System’ or ‘Metabolism’. Genes upregulated in mye(6 h)-macrophages versus untreated (M0) were highly enriched for the pathways ‘Immune System’, ‘Cytokine Signalling In Immune System’, ‘Interferon Signalling’ and ‘Interferon Alpha/Beta Signalling’. Even though the overall trend was similar in mye(6 h)-macrophages and activated M1-macrophages, the enrichment was more pronounced in the former. In contrast, genes enriched after 24 h myelin-treatment versus untreated (M0) in the pathways ‘Immune System’, ‘Innate Immune system’, ‘Cytokine Signalling in Immune System’, ‘Signalling By Interleukin’, ‘Interleukin-4 and Interleukin-13 Signalling’ and ‘Interleuking-10 Signalling’ were downregulated. Upregulated genes after prolonged myelin-uptake were mildly enriched for the pathways ‘Metabolism’, ‘Metabolism of Lipids’, and ‘Cholesterol Biosynthesis’, similarly to M2 macrophages, further confirming the PCA results (Fig. 3A; Supplementary Table 2).

To further characterize the phenotypes of the different macrophage populations, we performed ELISAs. As expected M1 polarized macrophages secreted TNFα, IFNγ as well as IL10, whereas M2 polarized macrophages released IL10, but no significant levels of TNFα, IL1β or IFNγ (Fig. 3B). Short-term myelin uptake induced TNFα (14 710 ± 1256 pg/ml; 43 754 ± 10 395), IL10 (2382 ± 592.4 pg/ml) and IL1β (18.48 ± 4.920 pg/ml), whereas mye(24 h) released high levels of IL1β (18.48 ± 4.920) and IL10 (5391 ± 545.2 pg/ml). Only M1-polarized, but not mye(6 h)- nor mye(24 h)-macrophages secreted significant IFNγ levels. Although not all of these changes reached statistical significance compared with naive M0 macrophages, which did not secrete detectable levels of TNFα, IL1β or IFNγ, the observed increases still represent a substantial induction of cytokine release. Latex bead controls verified myelin-specificity of observed effects (Fig. 3B)

Overall, initial uptake induced a pro-inflammatory phenotype that switched to anti-inflammatory after prolonged myelin exposure, correlating with lipid droplet formation.

### Oligodendrocyte differentiation is significantly impaired by mye-macrophage secretome despite phenotypic switch

To determine the impact of myelin-phagocytosing macrophages on oligodendrocytes, human induced (hi)OL were differentiated via doxycycline-inducible SOX10, OLIG2, NKX6.2 and small molecules over 23 days, yielding ∼50% O4^+^ and 25% MBP^+^ cells (Fig. 4A and B).^27^ Cell viability was unaffected by macrophage-conditioned medium, as shown by CellTiter-Glo analyses, which was further supported by quantification of DAPI^+^ nuclei (Fig. 4C and D). To determine the impact of macrophage supernatants on oligodendroglial differentiation, we quantified the proportion of O4^+^ and MBP^+^ oligodendrocytes. Medium controls, M0 and M2-polarized macrophage supernatants did not affect O4^+^ and MBP^+^ oligodendrocyte numbers, whereas mye(6 h)-, mye(24 h)- and M1-macrophage supernatants significantly reduced oligodendrocyte differentiation (Fig. 4E and F).

**Figure 4:**
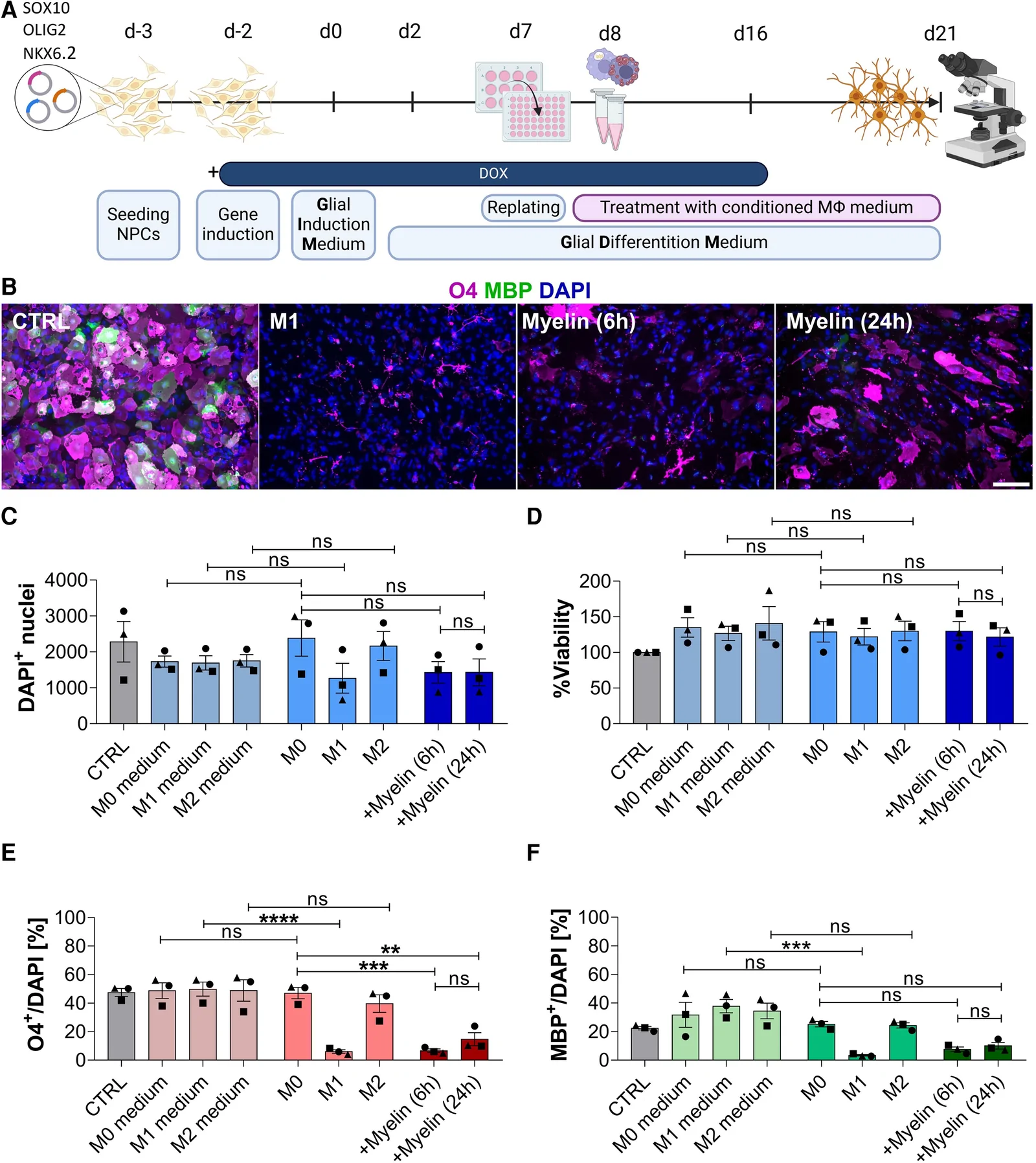
**Macrophage conditioned medium after myelin uptake severely impairs oligodendrocyte differentiation.** (**A**) Schematic of experimental design. Neural progenitor cells (NPCs) were differentiated into human-induced oligodendrocytes (hiOL) by the dox-inducible overexpression of the transcription factors SOX10, OLIG2, NKX6.2 and addition of small molecules within 23 days. On day 8, differentiation cells were treated with supernatants (1:5) from macrophages (Mɸ) that were either polarized into a pro-inflammatory (M1) or anti-inflammatory (M2) phenotype or treated with 12.5 µg myelin/250,000 cells for 6 h (initial myelin uptake) or 24 h (myelin-induced lipid droplet formation) till end of differentiation. Created in BioRender. Schmitz-Gielsdorf, L. (2026) https://BioRender.com/i5eh7au  **(B)** Representative immunocytochemistry (ICC) staining of treated and untreated hiOL for O4, myelin-basic protein (MBP) and DAPI. **(C)** Survival indicated by number of DAPI^+^ nuclei (F (DFn, DFd): F (1.409, 2.818) = 2.603) and **(D)** viability as measured by CellTiter-Glo Assay are not affected by treatment with macrophage conditioned medium (1:5) (F (DFn, DFd): F (1.464, 2.928) = 1.508). **(E)** Quantification of O4^+^ (F (DFn, DFd): F (8, 18) = 16.98) and **(F)** MBP^+^ cells after treatment with M1 or mye-macrophage supernatants showed significantly impaired differentiation (F (DFn, DFd): F (8, 18) = 9.407). Each symbol represents an NPC cell line as biological replicate, each with three technical replicates; three independent differentiations per cell line were performed. Mean ± SEM (*P < 0.05, **P < 0.01, ***P < 0.001, ****P < 0.0001; one-way ANOVA with Tukey’s). All comparisons are indicated in the graph; scale bar = 100 µm. DFd, degrees of freedom denominator; DFn, degrees of freedom numerator; DOX, doxycycline; F, f-value; MΦ, macrophage; NPC, neural progenitor cell.

This analysis showed that despite the observed phenotypic switch, both mye(6 h)- and mye(24 h)-macrophage-conditioned medium are detrimental for oligodendrocyte differentiation similarly to M1-macrophages.

### Neither C1q nor IL10 are responsible for impaired differentiation by mye(24 h)-macrophages

Next, we aimed to clarify which inflammatory mediators secreted by mye(6 h)-macrophages and mye(24 h)-macrophages contribute to impaired oligodendroglial differentiation. Since M1-, mye(6 h)- and mye(24 h)-macrophages all demonstrated a significant upregulation of IL10, we assessed whether increased levels of this cytokine may reduce oligodendroglial differentiation. We used concentrations matching those in diluted supernatants (0, 5, 1, 2 ng/ml) but observed no significant effect on oligodendrocyte differentiation (Supplementary Fig. 3).

To further narrow down potential candidates, we compared the transcriptomic profiles to M2-macrophages displaying no significant negative effect on oligodendroglial differentiation and mye(24 h)-macrophages. We selected the top 500 significantly differentially expressed genes, performed EnrichR analysis using the Reactome 2022 database and included all genes from the top ten enriched pathways. Interestingly, despite the clear detrimental effect of mye(24 h)-macrophages, numerous genes encoding factors known to benefit oligodendrocytes were upregulated (e.g. platelet-derived growth factor β, transforming growth factor α, activin A, vitronectin and galanin; Supplementary Table 3).^10,31-35^

Based on the literature, we identified a number of candidate genes potentially contributing to the reduced oligodendrocyte differentiation including complement factor C1q, cathepsins Z, V, B and C, matrix metalloproteinase-12 (MMP12) and fibronectin (Supplementary Table 3).^7,36-44^

Since C1q was shown to be upregulated in microglia in multiple sclerosis lesions and had been shown to have implications on oligodendrocyte differentiation and remyelination,^43,45^ we exposed oligodendrocytes to recombinant C1q (5, 10, 20 µg/ml) during differentiation; however, C1q had no significant effect on oligodendrocyte differentiation into either O4^+^ (65.21 ± 10.86% versus 69.78 ± 14.66%) or MBP^+^ cells (47.60 ± 11.11% versus 41.42 ± 7.30%) compared with control. Additionally, no significant impact on viability (88.52 ± 13.85%) and number of DAPI^+^ nuclei (5712 ± 671.8 versus 3980 ± 519.8) could be detected (Supplementary Fig. 4).

### Primary human microglia mirror macrophage response to myelin uptake

To assess whether blood-derived macrophages and brain-resident microglia differ in response to myelin uptake and its impact on oligodendrocytes, we repeated the experiments with primary human microglia. Cells were exposed to myelin for 6 and 24 h, paralleling macrophage conditions. ELISA of mye-microglia supernatants showed a significant TNFα increase at 6 h (4049 ± 1537 pg/ml) with low levels of IL1β and IL10 (0.77 ± 0.77 and 124.0 ± 70.62 pg/ml, respectively). After 24 h, TNFα decreased (1131 ± 261 pg/ml), whereas IL1β and IL10 increased (8.74 ± 5,75 and 1051 ± 416 pg/ml). IFNγ was undetectable at both time points (Fig. 5A). Similar to macrophages, supernatants from myelin-phagocytosing microglia impaired differentiation of human iPSC-derived NPC into O4^+^ or MBP^+^ oligodendrocytes in a dose-dependent manner, without affecting oligodendrocyte viability (Fig. 5B–E).

**Figure 5:**
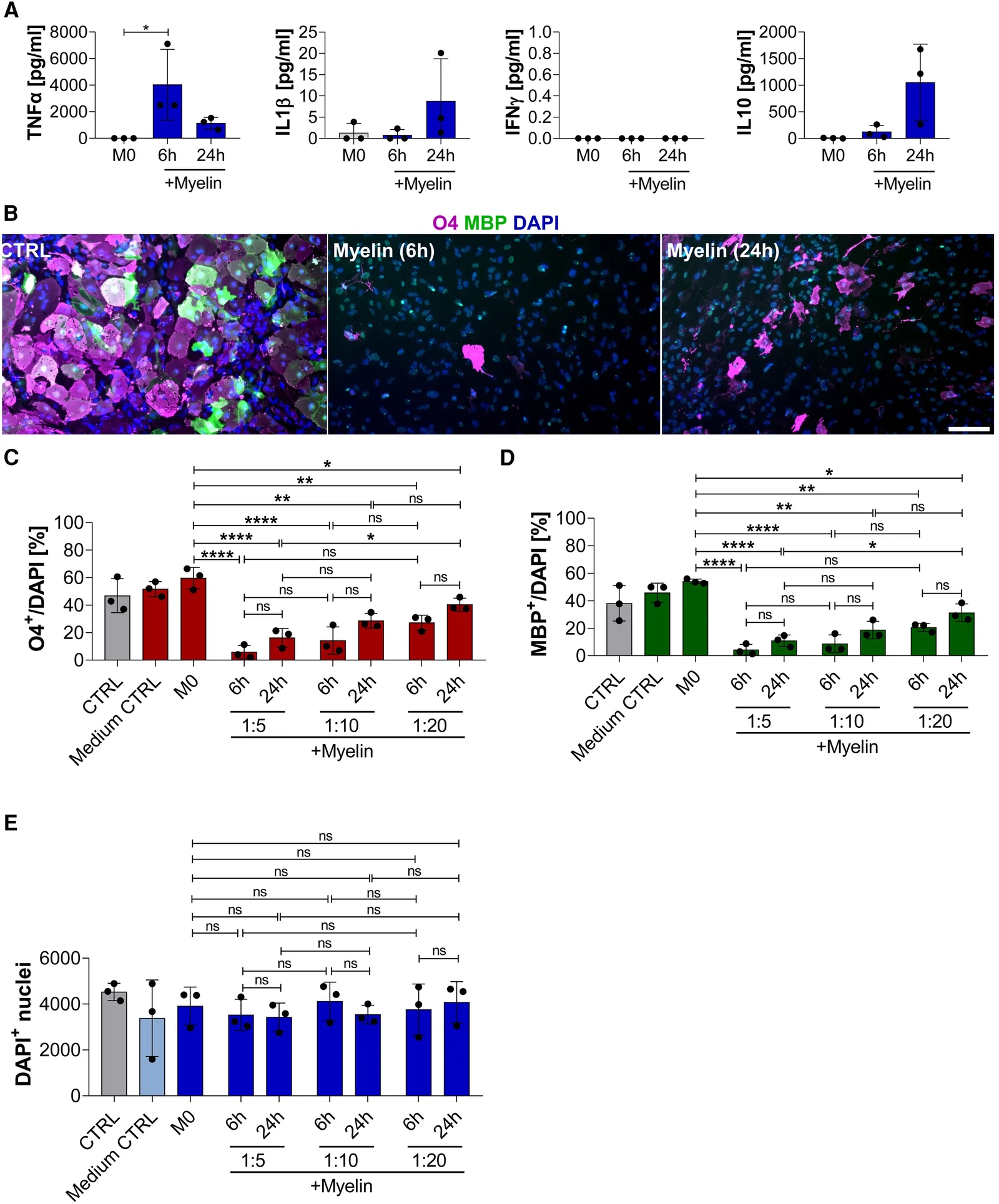
**Myelin-induced polarization of human primary microglia switches from initially pro-inflammatory to anti-inflammatory after prolonged uptake and inhibits oligodendrocyte differentiation.** (**A**) Analysis of mye(6 h)- and mye(24 h)-microglia (MG) conditioned medium via ELISAs for TNFα (F (DFn, DFd): F (2, 6) = 5.325), IL1β (F (DFn, DFd): F (2, 6) = 1.690), IFNγ and IL10 (F (DFn, DFd): F (2, 6) = 5.507). (**B**) Representative immunocytochemistry (ICC) staining of treated and untreated human-induced oligodendrocytes (hiOL) for O4, myelin-basic protein (MBP) and DAPI. (**C**) Quantification of O4^+^ (F (DFn, DFd): F (8, 18) = 18.69) and (**D**) MBP^+^ hiOL after treatment with mye-MG conditioned media showed significantly impaired differentiation in a dose-dependent manner (F (DFn, DFd): F (8, 18) = 21.55). (**E**) Survival as measured by number of DAPI^+^ nuclei is not affected by treatment with microglia conditioned medium (F (DFn, DFd): F (1.133, 2.265) = 0.8117). Three independent microglia differentiations with a total of three biological replicates for microglia and one biological replicate for hiOL were performed. Mean ± SEM (*P < 0.05, **P < 0.01, ***P < 0.001, ****P < 0.0001; One-way ANOVA with Tukey’s); all comparisons are indicated in the graph; scale bar = 100 µm. CTRL, control; DFd, degrees of freedom denominator; DFn, degrees of freedom numerator; F, f-value; MO, supernatant of untreated microglia.

Thus, similar to macrophages, microglia initially adopt a pro-inflammatory profile after myelin uptake, shift towards an anti-inflammatory signature with prolonged exposure, yet at both stages secrete factors, which impair oligodendroglial differentiation.

### Multiple sclerosis macrophages do not react to control myelin, but to multiple sclerosis myelin

Next, we asked whether macrophages from treatment naive relapsing-remitting multiple sclerosis (RRMS) patients behave like those from healthy individuals. multiple sclerosis macrophages phagocytosed myelin similarly to healthy controls, with MBP^+^ debris detectable from 3 h onwards and accumulating over time. LD formation, measured by the proportion of PLIN2^+^ macrophages, was significantly increased after 24 and 48 h after myelin uptake, similarly to healthy control macrophages (Fig. 6A and B, Fig. 1G and H). However, control myelin did not induce polarization of multiple sclerosis macrophages and oligodendrocyte differentiation remained unaffected (Fig. 6C–F). Successful M1 activation of multiple sclerosis macrophages and resulting cytokine secretion verified that multiple sclerosis macrophages could respond to pro-inflammatory polarizing stimuli, but not to control myelin, despite normal uptake and LD formation (Fig. 6C–F). Consistent with this, supernatants from myelin-laden multiple sclerosis macrophages had no effect on iPSC-derived oligodendrocyte viability (Fig. 6G).

**Figure 6:**
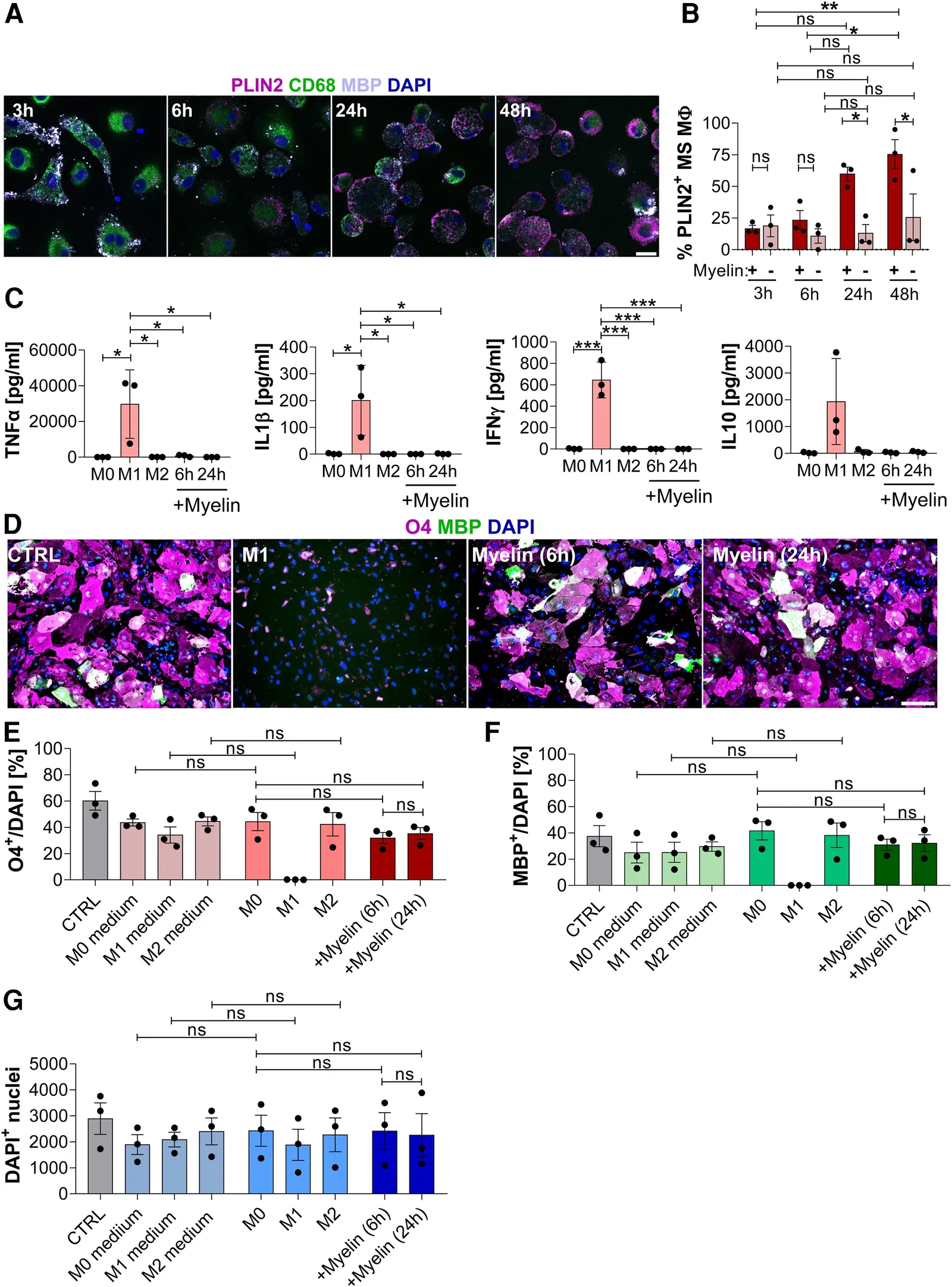
**Multiple sclerosis blood-derived macrophages show no cytokine secretion after myelin treatment and do not impair oligodendrocyte differentiation. (A)** Immunocytochemistry **(**ICC) of multiple sclerosis blood-derived macrophages (Mɸ) for pan-macrophage marker CD68, lipid droplet marker perilipin 2 (PLIN2), myelin marker myelin-basic protein (MBP) and nuclear marker DAPI at different timepoints of myelin treatment (3, 6, 24 and 48 h). **(B)** Quantification of respective ICC shows significant increase in lipid droplets after 24 and 48 h of myelin treatment (F (DFn, DFd): F (7, 16) = 6.331). **(C)** Analysis of supernatants from multiple sclerosis blood-derived macrophages (Mɸ) that were either polarized into a pro-inflammatory (M1) or anti-inflammatory (M2) phenotype or treated with myelin via ELISA revealed no significant induction of TNFα (F (DFn, DFd): F (4, 10) = 7.111), IL1β (F (DFn, DFd): F (4, 10) = 7.032), IFNγ (F (DFn, DFd): F (4, 10) = 44.59), IL10 (F (DFn, DFd): F (4, 10) = 4.151) secretion in response to myelin. **(D)** Representative ICC staining of untreated and treated human induced oligodendrocytes (hiOLs) for O4, MBP and DAPI. **(E)** Quantification of O4^+^ (F (DFn, DFd): F (1.938, 3.876) = 13.77) and **(F)** MBP^+^ cells after treatment showed that supernatants from multiple sclerosis macrophages after myelin-uptake did not affect oligodendrocyte differentiation (F (DFn, DFd): F (1.267, 2.534) = 5.394). **(G)** Survival of hiOL, as indicated by number of DAPI^+^ nuclei is not impaired after treatment with conditioned media of multiple sclerosis macrophages that phagocytosed control myelin (F (DFn, DFd): F (1.482, 2.964) = 1.140). Each analysis was performed with one independent experiment, with three biological replicates for multiple sclerosis macrophages *n* = 3 and one cell line as biological replicate for hiOL. Mean ± SEM (*P < 0.05, **P < 0.01, ***P < 0.001, ****P < 0.0001; one-way ANOVA with Tukey’s). For (**B, E, F** and **G**), all comparisons are indicated in the graphs; for (**C**), each condition was compared to each other condition; only significant differences are depicted in the graphs. Scale bars in A = 20 µm and in D = 100 µm. CTRL, control; DFd, degrees of freedom denominator; DFn, degrees of freedom numerator; F, f-value.

To examine whether the absent cytokine response to control myelin is specific to multiple sclerosis macrophages, we treated macrophages from stroke and autoimmune encephalitis (AE) patients with control myelin and analysed cytokine secretion and effects on oligodendrocyte differentiation. Similar to multiple sclerosis macrophages, both stroke and AE macrophages secreted no significant levels of TNFα, IL1β, IFNγ or IL10 (Fig. 7A to B) and conditioned media did not impair oligodendrocyte differentiation (Fig. 7C to J).

**Figure 7:**
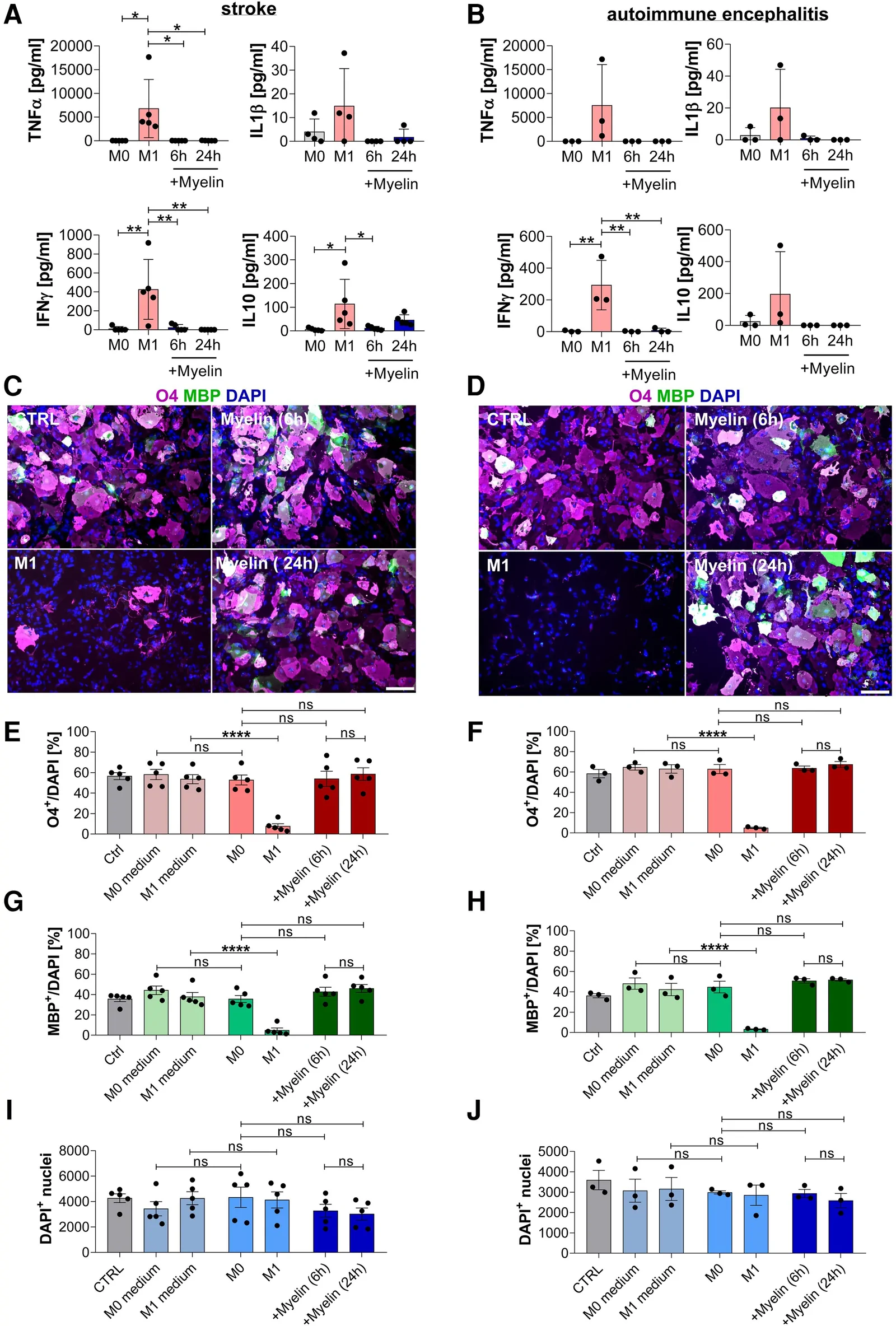
**Blood-derived macrophages from stroke and autoimmune encephalitis patients show no cytokine secretion after myelin treatment and do not impair oligodendrocyte differentiation.** (**A, B**) Analysis of supernatants of blood-derived macrophages (Mɸ) from stroke and autoimmune encephalitis (AE) patients via ELISA revealed no significant induction of TNFα (F (DFn, DFd): F (3, 16) = 6.125, F (3, 8) = 2.350), IL1β (F (DFn, DFd): F (3, 12) = 2.457, F (3, 8) = 1.781) IFNγ (F (DFn, DFd): F (3, 16) = 8.498, F (3, 8) = 10.24), IL10 (F (DFn, DFd): F (3, 16) = 4.269, F (3, 8) = 1.491) secretion after myelin uptake. (**C, D**) Representative immunocytochemistry (ICC) stainings of treated and untreated human-induced oligodendrocytes (hiOL) for O4, myelin-basic protein (MBP) and DAPI. **(E, F)** Quantification of O4^+^ (F (DFn, DFd): F (6, 28) = 13.09, F (6, 14) = 45.95) and **(G, H)** Myelin-basic protein-positive (MBP^+^) cells after treatment showed that supernatants from macrophages from stroke and AE patients after myelin-uptake did not affect oligodendrocyte differentiation (F (DFn, DFd): F (6, 28) = 14.55, F (6, 14) = 17.96). **(I, J)** Oligodendrocyte survival, as indicated by number of DAPI^+^ nuclei was not affected by stroke or AE macrophage supernatants (F (DFn, DFd): F (2.090, 8.359) = 2.503, F (1.943, 3.886) = 0.4741). Each analysis was performed with two independent experiments, with a total of five donors as biological replicates for stroke macrophages, three donors as biological replicates for AE macrophages, one NPC cell line as a biological replicate for hiOL. Mean ± SEM. (*P < 0.05, **P < 0.01, ***P < 0.001, ****P < 0.0001; one-way ANOVA with Tukey’s). For (**A, B**), each condition was compared with each other condition; only significant differences are depicted in the graphs; for (**E–J**) all comparisons are indicated in the graph; scale bars in A = 15 µm, C and D = 100 µm. CTRL, control; DFd, degrees of freedom denominator; DFn, degrees of freedom numerator; F, f-value; naive: M0, pro-inflammatory (M1).

We then tested whether multiple sclerosis myelin could overcome this ‘silenced phenotype’. Since media controls consistently had no effect on OL differentiation and the number of macrophages from treatment naive RRMS donors were limited, we decided to omit technical replicates for media controls (*n* = 1) (Fig. 7E to G). All other relevant conditions were performed in triplicate (*n* = 3). Multiple sclerosis myelin was phagocytosed as efficiently as control myelin by multiple sclerosis macrophages and also significantly induced LD formation after 24 and 48 h (Fig. 8A and B). Unlike control myelin, however, multiple sclerosis myelin elicited cytokine release upon myelin phagocytosis comparable to that induced by control myelin in healthy macrophages (Fig. 8C to G, Fig. 4B to F). After 6 h, supernatants of multiple sclerosis myelin-laden macrophages contained TNFα (6577 ± 1528 versus 14 710 ± 1256 pg/ml) and IL10 (4787 ± 295.1 versus 2382 ± 592.4 pg/ml). After 24 h, TNFα decreased (805 ± 403.8 pg/ml), IL1β increased (40.20 ± 19.01 pg/ml) and IL10 remained high (4619 ± 226.2 pg/ml) (Fig. 8C). Although not all of these changes reached statistical significance compared with naive M0 macrophages, the observed increases still represent a substantial induction of cytokine release. In line with these findings, both mye(6 h) and mye(24 h) supernatants significantly impaired oligodendroglial differentiation (Fig. 8D to G).

**Figure 8:**
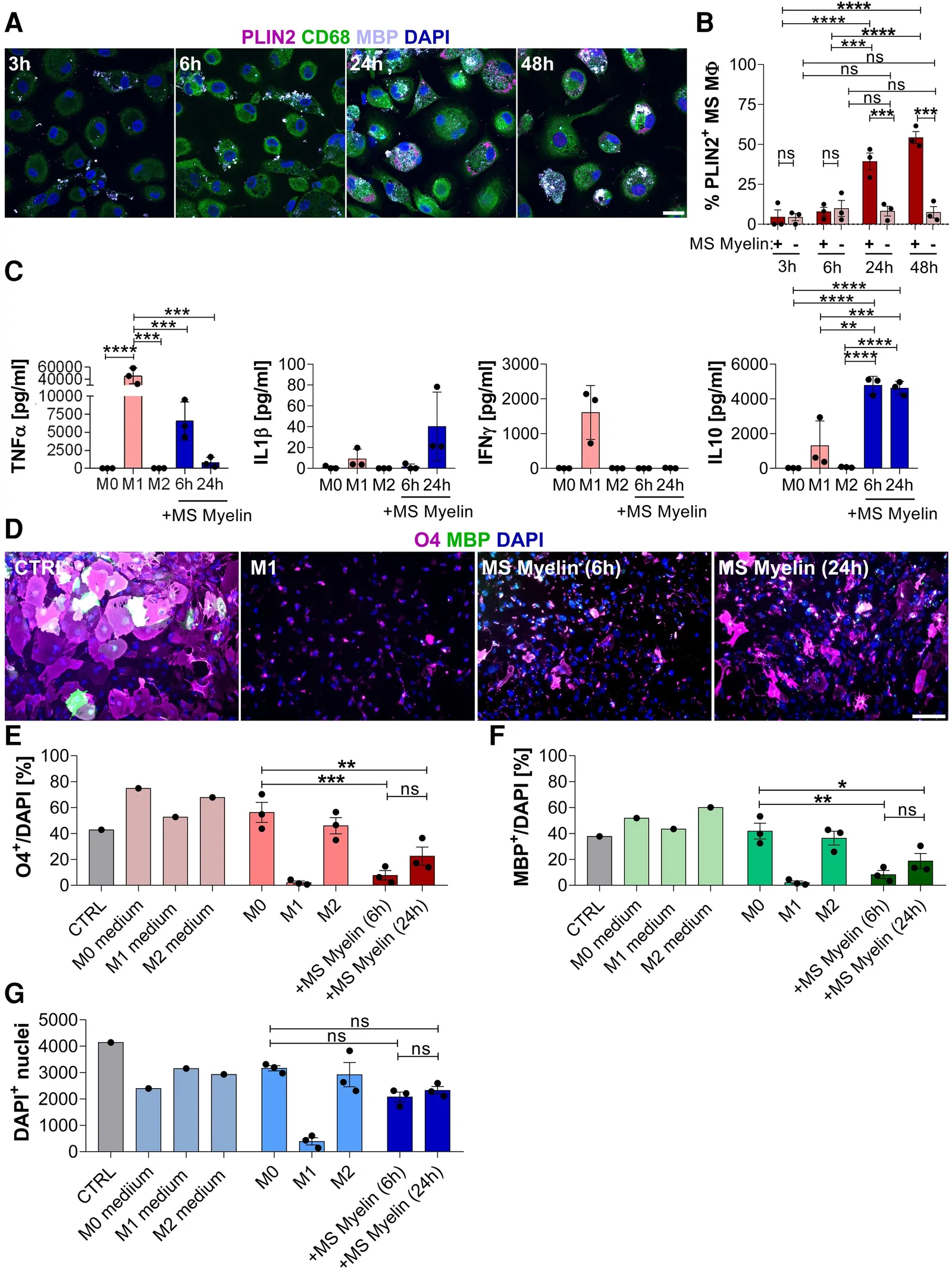
**Multiple sclerosis myelin does elicit polarization of multiple sclerosis macrophages and causes impairment of OL differentiation.** (**A**) Immunocytochemistry **(**ICC) of multiple sclerosis blood-derived macrophages (Mɸ) for pan-macrophage marker CD68, lipid droplet marker perilipin 2 (PLIN2), myelin marker myelin-basic protein (MBP) and nuclear marker DAPI at different timepoints of multiple sclerosis myelin treatment (3, 6, 24 and 48 h). (**B**) Quantification of respective ICC shows significant increase in lipid droplets after 24 and 48 h of multiple sclerosis myelin treatment (F (DFn, DFd): F (7, 16) = 23.49). (**C**) Analysis of supernatants via ELISA for TNFα (F (DFn, DFd): F (4, 10) = 31.82), IL1β (F (DFn, DFd): F (4, 10) = 3.715), IFNγ (F (DFn, DFd): F (4, 10) = 12.82) and IL10 (F (DFn, DFd): F (4, 10) = 35.11). (**D**) Representative ICC staining of untreated and treated hiOL for O4, MBP and DAPI. (**E**) Quantification of O4^+^ (F (DFn, DFd): F (8, 10) = 13.74) and (**F**) MBP^+^ cells after treatment showed that supernatants from multiple sclerosis macrophages after multiple sclerosis myelin-uptake do impair oligodendrocyte (OL) differentiation regardless of phenotypic switch (F (DFn, DFd): F (8, 10) = 11.55). (**G**) Survival of human-induced OL (hiOL), as indicated by the number of DAPI^+^ nuclei is not impaired after treatment with conditioned media of multiple sclerosis macrophages that phagocytosed multiple sclerosis myelin (F (DFn, DFd): F (8, 10) = 13.35). Each analysis was performed with one independent experiment, with three biological replicates for multiple sclerosis macrophages, one NPC cell line as a biological replicate for hiOL. Mean ± SEM (*P < 0.05, **P < 0.01, ***P < 0.001, ****P < 0.0001; one-way ANOVA with Tukey’s, F = f-value, DFn = degrees of freedom numerator, DFd: degrees of freedom denominator). For (**B, E, F, G**), all comparisons are indicated in the graph; for (**C**), each condition was compared to each other condition; only significant differences are depicted in the graphs. Sale bar in A = 20 µm and in D = 100 µm. CTRL, control; DFd, degrees of freedom denominator; DFn, degrees of freedom numerator; F, f-value; naive, M0, pro-inflammatory (M1), anti-inflammatory (M2).

In summary, our findings suggest that only multiple sclerosis myelin, but not control myelin, activates macrophages from individuals with CNS diseases, resulting in impaired oligodendroglial differentiation.

### Human control myelin induces limited activation of stroke macrophages, whereas multiple sclerosis myelin elicits a stronger response

Because murine myelin was used in earlier experiments due to limited human material and its similarity to human myelin, we repeated experiments with healthy human control myelin. Given the scarcity of therapy-naive RRMS macrophages and the significant effect of multiple sclerosis myelin on RRMS macrophages already shown in Fig. 8, stroke macrophages were used. Uptake of healthy human control myelin, induced TNFα and IL10 after 6 and 24 h (TNFα: 1071 ± 314.3 and 295.1 ± 5.412 pg/ml; IL10: 307.4 ± 195.6 and 551.2 ± 268.5 pg/ml). In contrast, multiple sclerosis myelin triggered markedly higher levels of TNFα and IL10 (TNFα: 4193 ± 845.3 and 3854 ± 791.0 pg/ml; IL10: 259.2 ± 165.8 and 2421 ± 1235 pg/ml; (Fig. 9A and B); however, due to higher variability, no statistical significance was reached.

**Figure 9:**
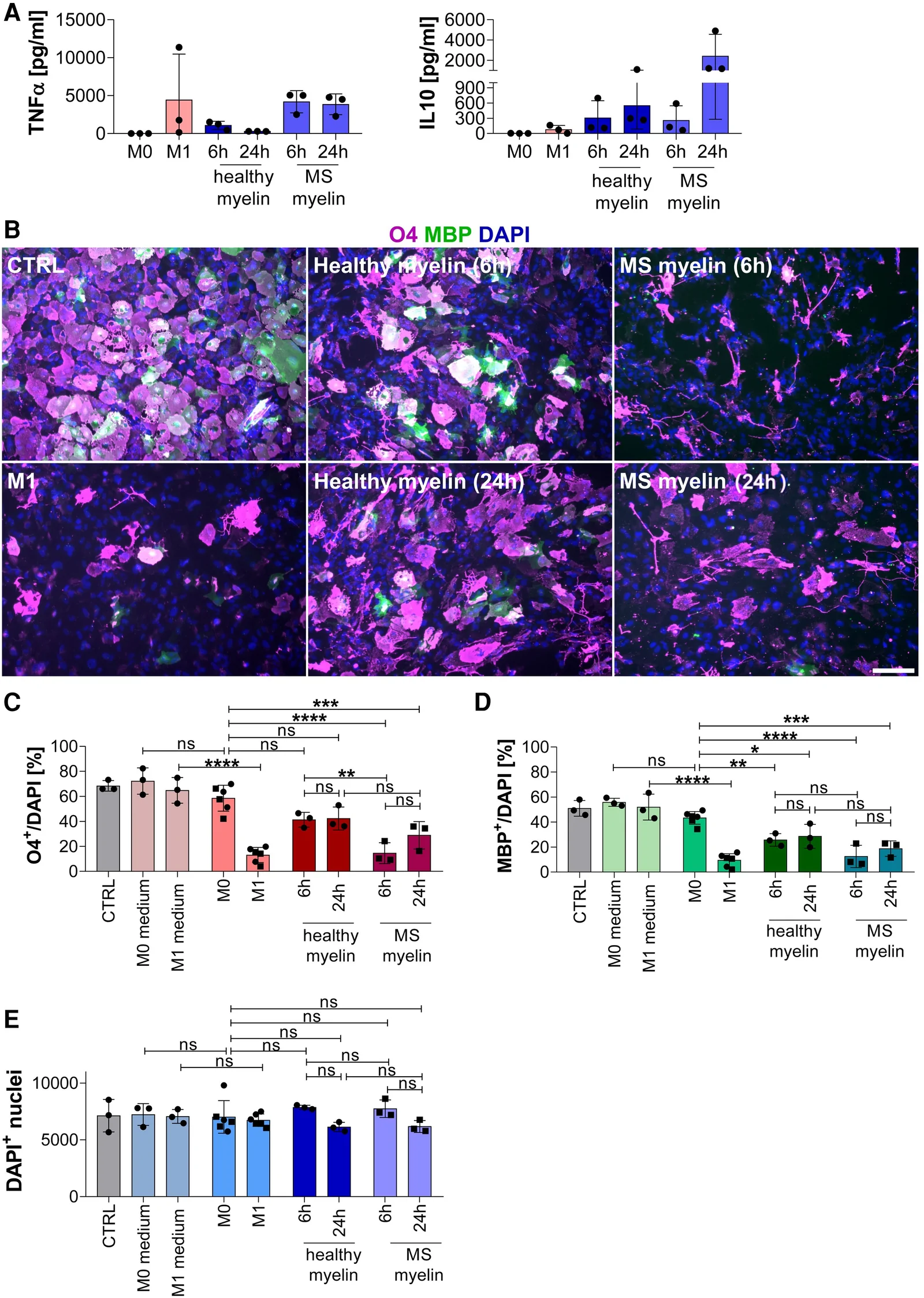
**Human control myelin exerts less impact on oligodendrocyte differentiation than MS myelin.** (**A**) Analysis of supernatants of blood-derived macrophages (Mɸ) from stroke patients via ELISA revealed lower TNFα (F (DFn, DFd): F (5, 12) = 1.874) and IL10 levels (F (DFn, DFd): F (5, 12) = 2.988) after uptake of human control myelin compared to multiple sclerosis myelin. (**B**) Representative immunocytochemistry (ICC) stainings of untreated and treated human induced oligodendrocytes (hiOL) for O4, myelin-basic protein (MBP) and DAPI. (**C**) Quantification of O4^+^ (F (DFn, DFd): F (8, 24) = 26,15) and **(D)** MBP^+^ cells treated with supernatants from macrophages phagocytosing MS myelin or myelin from healthy individuals (F (DFn, DFd): F (8, 24) = 27.44). **(E)** Oligodendrocyte survival, as indicated by number of DAPI^+^ nuclei was not affected by stroke macrophages after uptake of human control or multiple sclerosis myelin (F (DFn, DFd): F (9, 24) = 1.332). Each analysis was performed with one independent experiment, three biological replicates for stroke macrophages, one biological replicate (cell line) for hiOL. Mean ± SEM. (*P < 0.05, **P < 0.01, ***P < 0.001, ****P < 0.0001; one-way ANOVA with Tukey’s); for (**A**), each condition was compared to each other condition; only significant differences are depicted in the graphs; for **(C–E**), all comparisons are indicated in the graph. Scale bar in **B** = 100 µm. CTRL, control; DFd, degrees of freedom denominator; DFn, degrees of freedom numerator; F, f-value; naive, M0, pro-inflammatory (M1).

Supernatants from healthy myelin-laden stroke macrophages caused only a mild, statistically non-significant reduction in O4^+^ oligodendrocytes but significantly impaired terminal differentiation into MBP^+^ macrophages. In contrast, supernatants from multiple sclerosis myelin supernatants (mye(6 h), mye(24 h)), strongly reduced both O4^+^ and MBP^+^ oligodendrocytes (Fig. 9C–E).

In summary, our data suggest that while species-specific effects were observed, multiple sclerosis myelin consistently exerted a stronger impact on macrophage activation and impaired oligodendrocyte differentiation compared to healthy human myelin. To identify proteins potentially driving the increased immune response to multiple sclerosis versus control myelin, we performed mass spectrometry on the same myelin preparations used *in vitro*. In total, 5342 proteins were detected, of which 722 were ≥2-fold higher in multiple sclerosis sample and selected for further analysis. Filtering for immune- or multiple sclerosis-associated proteins highlighted several candidates. Literature review identified four of particular relevance: fibrinogens (FGA, FGB and FGG), high mobility group box 1 and 2 (HMGB1/2), chitinase-3-like protein 1 (CHI3L1) and citrullinated MBP (Supplementary Tables 5 and 6). All of these have been previously reported to be elevated in multiple sclerosis and linked to heightened pro-inflammatory effects.

## Discussion

In multiple sclerosis, macrophages and microglia, key players in both inflammation and repair, take up myelin degradation products. While clearance is essential for remyelination, its impact on phagocytes and the lesion milieu remains unclear. Impaired lipid metabolism in these cells has been linked to multiple sclerosis pathology, with animal studies suggesting that myelin-induced lipid accumulation disrupts phagocytosis, increases reactive oxygen species and pro-inflammatory cytokines and hampers repair.^46^ Yet validation in human systems is lacking. Our study addresses this gap, by examining lipid droplet formation and myelin phagocytosis using human cells and multiple sclerosis tissue samples. We demonstrate a dynamic inflammatory response in human macrophages and microglia following myelin uptake. Despite a shift from pro- to anti-inflammatory phenotypes, both states inhibited OL differentiation and we provide candidates potentially contributing to this detrimental effect. Moreover, multiple sclerosis myelin elicited stronger inflammatory response in macrophages, suggesting that it acts as an immunogenic driver that disrupts a hypothesized immune tolerance and impairs regeneration.

In rodent demyelination models, aged phagocytes accumulate excessive myelin debris and fail to resolve inflammation, impairing remyelination.^20^ This led to the hypothesis that cholesterol overload, reflected by LD formation, may contribute to remyelination failure in multiple sclerosis. However, although PLIN2^+^ LDs are a characteristic feature of myelin-laden macrophages in active multiple sclerosis lesions, we did not observe a correlation between PLIN2^+^ LDs in myeloid cells and impaired remyelination in multiple sclerosis lesions.

The phenotype of myelin-laden macrophages/microglia has been inconsistently described, reflecting experimental heterogeneity and rodent predominance. Some studies report a pro-inflammatory phenotype after myelin uptake, while others observe anti-inflammatory or no responses.^15,16,47-50^ Lipid-laden ‘foamy’ macrophages in active multiple sclerosis lesions are often considered anti-inflammatory, whereas rodent models associate lipid overload to a pro-inflammatory phenotype and impaired repair, based on the observations that interventions enhancing lipolysis, lipophagy or cholesterol efflux restore remyelination.^4,20,21,51,52^

Our data show a biphasic inflammatory response in human macrophages/microglia after myelin uptake, with an initial pro-inflammatory phase shifting to an allegedly anti-inflammatory phenotype as LDs form. The detrimental effect of pro-inflammatory phagocytes has been shown before^5,9,53^; however, in our experiments, both phenotypes impaired OL differentiation, contrasting with rodent studies where M2-like macrophages support repair.^10^ Our data demonstrate that an M2-like or anti-inflammatory macrophage phenotype is not necessarily associated with neutral or beneficial effects on oligodendrocytes and further support the concept of highly dynamic and complex macrophages phenotype changes in the disease context.

Based on our results from the transcriptomic analyses and published literature, we identified candidate mediators that may inhibit OL differentiation, including C1q, cathepsins (C, Z, V and B) and matrix metalloproteinase-12 (MMP12). C1q, highly expressed in multiple sclerosis lesion rims and linked to ‘microglia inflamed in multiple sclerosis’ (MIMS), impairs OL differentiation in murine models, but had no effect in our human assays.^43,45^ Cathepsins, particularly cathepsin C, are implicated in multiple sclerosis pathology, with elevated expression and activity in experimental autoimmune encephalomyelitis and multiple sclerosis patients, while their inhibition improved outcomes in animal models.^40-42,54-59^ Similarly, MMP12 is associated with OL death, demyelination and macrophage recruitment in rodent models.^36,37^ Further work is required to confirm their role in human systems.

Macrophages from treatment-naive RRMS, stroke, and AE patients exposed to control myelin showed no polarization or hampered OL impairment, suggesting a downregulation of macrophage activity in the context of ongoing inflammatory stimulation and independent from the nature of the stimulus. This phenotype functionally resembles the phenotype of immune-paralytic monocytes and macrophages known from the context of acute systemic inflammation. This mechanism preserves the system from overshooting innate immune responses, but also harbours the risk of impaired immune responses following secondary infections.^60,61^

This phenomenon of a dampen immune response was shown to be epigenetically regulated. For example, after LPS training, murine microglia show reduced cytokine production upon re-exposure, linked to histone modifications.^62^ Similarly, myelin-laden macrophages display suppressed production of pro-inflammatory mediators such as NO and IL6 after LPS stimulation, indicating a potential myelin-induced immune tolerance.^16,49^ Epigenetic analysis of murine myelin-laden macrophages revealed decreased chromatin accessibility and reduced gene expression in inflammation-related pathways, contributing to an immunosuppressive phenotype.^63^ Together these findings suggest that also the lack of cytokine secretion in response to myelin phagocytosis by macrophages from individuals with multiple sclerosis, stroke, and AE observed in our study reflects immune paralysis potentially via epigenetic modulation.

Notably, multiple sclerosis–derived myelin overcame the immune-paralytic phenotype of macrophages, consistently eliciting stronger cytokine responses and greater impairment of OL differentiation compared to control myelin. These findings suggest that multiple sclerosis myelin represents either a quantitatively stronger or qualitatively different stimulus to phagocytes as compared to control myelin. Interestingly, primary human microglia and THP-1 macrophages phagocytose multiple sclerosis myelin more efficiently than control myelin.^25^ In line with these observations, we identified candidate proteins in our multiple sclerosis myelin preparation that are known to promote immune activation and may underlie the restored response of disease associated macrophages as observed in our assays. Fibrinogen, which deposits after blood–brain barrier disruption and induces microglial activation, leukocyte recruitment, and demyelination, thereby acting as a potent pro-inflammatory signal.^64-67^ Similarly, HMGB1/2, which can bind myelin and function as danger-associated molecular pattern, is elevated in multiple sclerosis and amplify inflammation through Toll-like receptors and RAGE.^68-71^ CHI3L1, highly expressed in multiple sclerosis tissue, further promotes immune activation via cytokine-driven signalling.^72,73^ Beyond these proteins, post-translational modifications of myelin also appear relevant. Peptidyl arginine deaminase–mediated citrullination of MBP destabilizes myelin structure and correlates with lesion activity, disease severity, and impaired remyelination in multiple sclerosis.^74-79^ Importantly, citrullinated MBP is preferentially recognized by T cells, promotes Th17 differentiation, exposes immunodominant epitopes, and provokes antibody responses.^80-84^ However, further studies are required to validate the role of these candidate proteins in eliciting a strong activation even in immune-paralytic macrophages.

In summary, our data demonstrate that remyelination failure in multiple sclerosis is not associated with the presence of PLIN2^+^ LDs in myeloid cells in active multiple sclerosis lesions. Instead, *in vitro* myelin-laden macrophages despite transitioning from a pro- to anti-inflammatory phenotype inhibit OL differentiation independent of LD formation. Moreover, disease associated macrophages exhibited an immune paralytic phenotype with reduced responses to control myelin, which could be overcome by multiple sclerosis myelin. Thus, our data challenge the paradigm that macrophages with an anti-inflammatory phenotype as characterized by cytokine and transcriptomic profiles promote repair and supports the concept that multiple sclerosis macrophages acquire an epigenetic memory, with multiple sclerosis myelin acting as a potent immunogenic mediator of failed regeneration.

## Supplementary Material

fcag411_Supplementary_Data

## Data Availability

The RNA-seq raw data generated in this study have been deposited in the NCBI Sequence Read Archive (SRA) under the BioProject accession number PRJNA1359350. The mass spectrometry proteomics data have been deposited to the ProteomeXchange Consortium via the PRIDE repository^31^ with the dataset identifier PXD068996.
