# Supplementary material for: Impact of myelin phagocytosis on human myeloid cells and its effect on human oligodendrocytes
Source: Brain Commun. 2026 Nov 3;8(6):fcag411. doi: 10.1093/braincomms/fcag411 (PMC13748744; doi:10.1093/braincomms/fcag411)
Supplement: Supplementary file 1 [file fcag411_supplementary_data.zip › Supplementary_material.pdf]

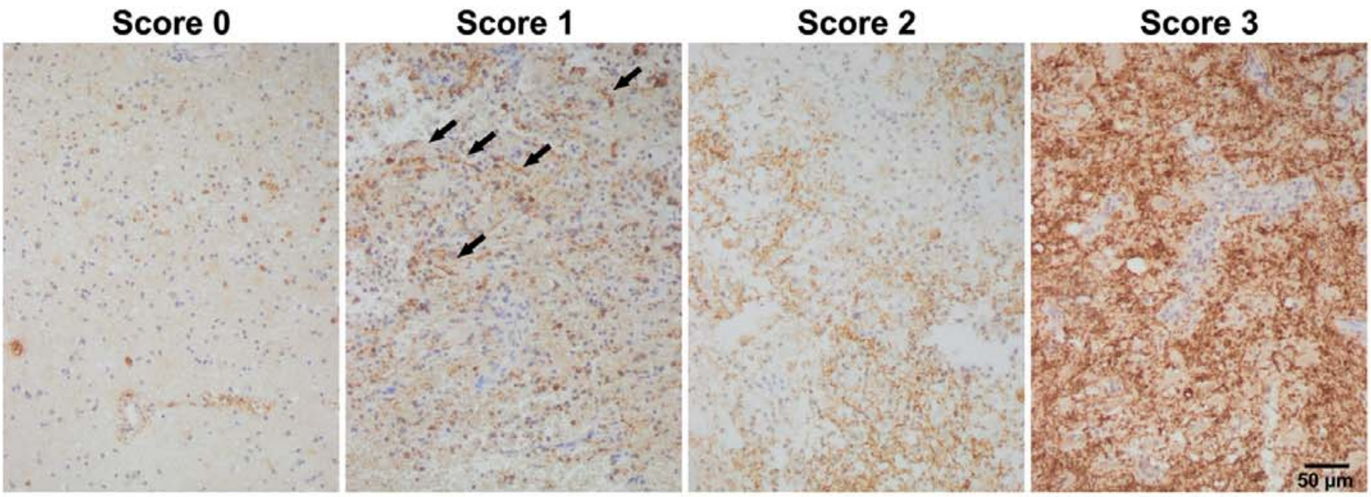

Supplementary Figure 1:
Remyelination score

Example images illustrating the different remyelination scores identified utilizing immunohistochemisty for myelin basic protein. scale bar = 50µm.

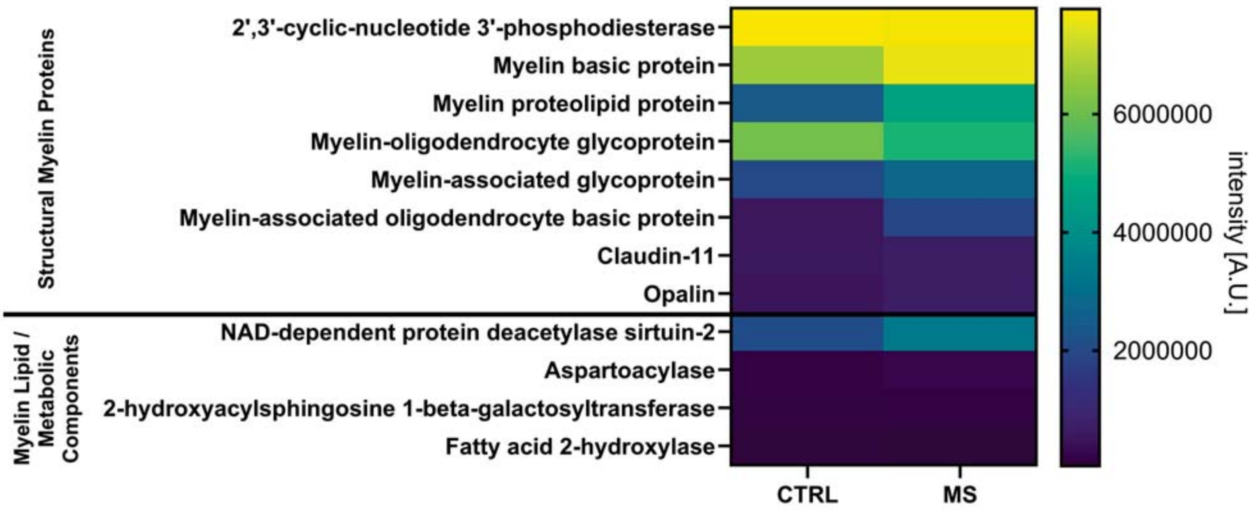

Supplementary Figure 2:
Control and MS myelin abundance of key myelin peptides
Heatmap displaying the plotted intensities of myelin structure components and lipids in the used control and MS myelin samples. A.U. = arbitrary unit

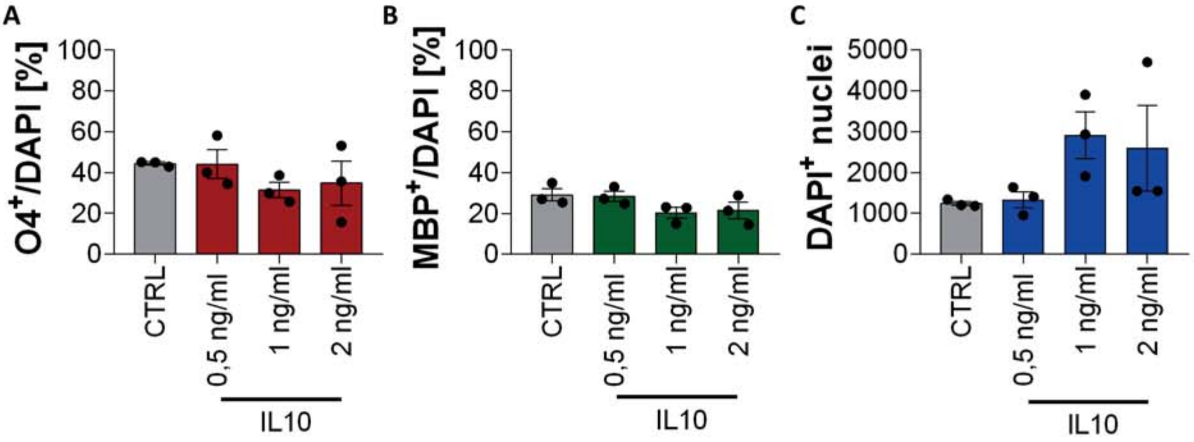

Supplementary Figure 3:
IL10 does not affect oligodendrocyte differentiation or viability.

(A) Quantifications of O4+ (F (DFn, DFd): F (4, 10) = 2,150) and (B) myelin-basic protein-positive (MBP+) cells (F (DFn, DFd): F (4, 10) = 1,853) show no direct effect of IL10 (0,5, 1, 2 ng/ml) on oligodendrocyte differentiation. (C) Survival indicated by number of DAPI+ nuclei (F (DFn, DFd): F (4, 10) = 3,527) are not affected by treatment with IL10. Three independent experiments with one biological replicate (cell line) were performed. Mean ± SEM (*P<0.05, **P<0.01, ***P<0.001, ****P<0.0001; One-way ANOVA with Dunnett`s, F= f-value, DFn = degrees of freedom numerator, DFd: degrees of freedom denominator); each condition was compared to CTRL; only significant

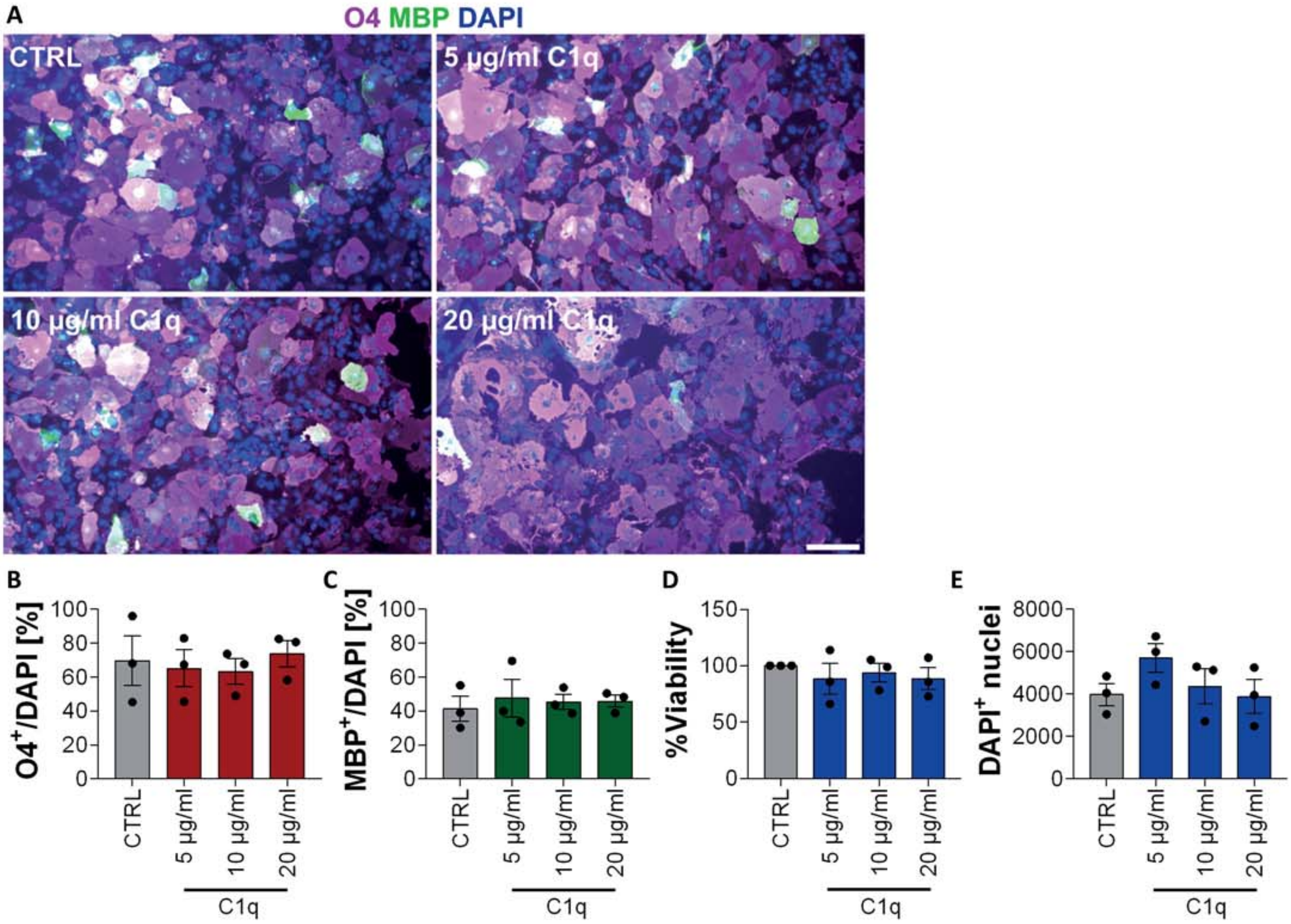

Supplementary Figure 4:
C1q does not affect oligodendrocyte differentiation or viability.

(A) Representative immunocytochemistry (ICC) staining of treated and untreated human induced oligodendrocyte (hiOL). (B) Quantifications of O4+ (F (DFn, DFd): F (1,027, 2,053) = 1,327) and (C) myelin-basic protein-positive (MBP+) cells (F (DFn, DFd): F (1,045, 2,091) = 0,4712) show no direct effect of complement factor C1q (5, 10, 20 µg/ml) on oligodendrocyte differentiation. (D) Viability as measured by CellTiter-Glo Assay (F (DFn, DFd): F (1,281, 2,561) = 2,533) and (E) survival indicated by number of DAPI+ nuclei are not affected by treatment with C1q (F (DFn, DFd): F (3, 8) = 1,377). Three independent experiments with one biological replicate (cell line) were performed. Mean ± SEM (*P<0.05, **P<0.01, ***P<0.001, ****P<0.0001; One-way ANOVA with Tukey's, F= f-value, DFn = degrees of freedom numerator, DFd: degrees of freedom denominator); each condition was compared to CTRL; only significant differences are depicted in the graphs. Scale bar in A = 100 µm

Supplementary Table 1: List of patient derived PBMCs

<table>
<tr><th>Disease</th><th>ID</th><th>Sex</th><th>Age</th><th>Cell type</th><th>Treatment</th><th>Myelin treatment</th></tr>
<tr><td rowspan="6">RRMS</td><td>08535</td><td>female</td><td>30</td><td rowspan="6">PBMCs</td><td rowspan="6">relapse therapy-naive</td><td rowspan="3">Human MS myelin</td></tr>
<tr><td>10029</td><td>male</td><td>58</td></tr>
<tr><td>10611</td><td>female</td><td>28</td></tr>
<tr><td>2241_1_C</td><td>male</td><td>55</td><td rowspan="3">Murine control myelin</td></tr>
<tr><td>10572_1_C</td><td>female</td><td>48</td></tr>
<tr><td>111_66_1_C</td><td>female</td><td>56</td></tr>
<tr><td rowspan="3">Autoimmune encephalitis</td><td>8365_1_C</td><td>male</td><td>70</td><td rowspan="8">PBMCs</td><td rowspan="8">-</td><td rowspan="8">Murine control myelin</td></tr>
<tr><td>6683_1_C</td><td>male</td><td>61</td></tr>
<tr><td>8013_1_C</td><td>female</td><td>68</td></tr>
<tr><td rowspan="11">Stroke</td><td>9305_2_C</td><td>female</td><td>92</td></tr>
<tr><td>9932_4_C</td><td>male</td><td>72</td></tr>
<tr><td>10723_1_C</td><td>male</td><td>70</td></tr>
<tr><td>10869_1_C</td><td>male</td><td>74</td></tr>
<tr><td>10975_1_C</td><td>female</td><td>74</td></tr>
<tr><td>10726_1_C</td><td>male</td><td>61</td><td rowspan="6">PBMCs</td><td rowspan="6">-</td><td rowspan="3">Human control myelin</td></tr>
<tr><td>10864_1_C</td><td>male</td><td>53</td></tr>
<tr><td>10958_1_C</td><td>female</td><td>77</td></tr>
<tr><td>10727_1_C</td><td>female</td><td>66</td><td rowspan="3">Human MS myelin</td></tr>
<tr><td>11021_1_C</td><td>female</td><td>71</td></tr>
<tr><td>8116-2_C</td><td>female</td><td>29</td></tr>
</table>

Supplementary Table 2: Overview of -Log10 (p-value) of most prominent enriched pathways in myelin-laden, M1 and M2 macrophages, after EnrichR analysis using the Reactome2022 database.

	M1	mye(6h)	M2	mye(24h)
Immune System	5,31	19,49	22,53	19,41
Cytokine Signalling In Immune System	8,21	24,71	24,2	20,18
Interferon Signalling	11,49	26,29	-	-
Interferon Alpha/Beta Signalling	9,7	27,67	-	-
Innate Immune System	-	-	7,93	9,44
Signalling By Interleukin	-	-	24,78	19,35
Interleukin-4 and Interleukin-13 Signalling	-	-	12,98	11,42
Interleukin-10 Signalling	-	-	17,23	12,76
Metabolism	-	-	4,31	3,35
Metabolism of Lipids	-	-	7,28	3,88
Cholesterol Biosynthesis	-	-	7,25	4,82

Supplementary Table 3: Top Enriched Reactome pathways and associated genes in mye(24h) vs. M2 macrophages (EnrichR, Reactome2022)

Term	Overlap	P-value	Adjusted P-value	Odds Ratio	Combined Score	Genes
Upregulated genes						
Immune System R-HSA-168256	58/1943	5,64E-06	0,002681137	2	24	CD86; CD40; CDKN1A; TNFAIP6; CLEC10A; CD80; CTSZ; CLSTN1; F13A1; CTSV; AP2A2; EEA1; PLAU; ANPEP; MRC1; QSOX1; CTSC; AP2M1; CTSB; EGFL7; CISH; IL1R2; AP1B1; RHOF; PDCD1LG2; MMP12; FCER2; CD200R1; SLC7A8; IRF4; CD226; VCL; CLEC4G; C1QB; CD274; MAOA; ALOX15; FGL2; CD1C; CD1B; LILRA3; CD79A; VTN; BCL2L11; SOCS1; CCL4; CCL3; CCL17; N4BP1; CCL22; SIGLEC10; FN1; SMAD7; CD209; TREML2; PTPN7; FCGR2B; HLA-DRB1
Interleukin-10 Signaling R-HSA-6783783	7/45	8,11E-06	0,002681137	11	132	CD86; FCER2; CCL22; CD80; IL1R2; CCL4; CCL3
Miscellaneous Transport And Binding Events R-HSA-5223345	5/23	3,08E-05	0,005233539	17	175	NIPAL1; NIPA1; CTNS; LRRC8A; LRRC8B
Immunoregulatory Interactions Between A Lymphoid And A non-Lymphoid Cell R-HSA-198933	10/123	3,67E-05	0,005233539	5	55	CD40; CD200R1; SIGLEC10; TREML2; CD226; CD1C; FCGR2B; CD1B; LILRA3; CLEC4G

Signal Transduction R-HSA-162582	66/2465	3,96E-05	0,005233539	2	18	CD86; CDKN1A; FLT1; CD80; CLSTN1; SPINT2; AP2A2; PCSK5; AKAP12; ITGB8; CCR7; GIT1; AP2M1; DACT1; TLE1; WNT5B; GPR35; FST; WNT5A; RHOF; TRAF1; F3; ARL4C; FCER2; CD200R1; GAL; SPINT1; DAAM1; GFOD1; ALDH1A2; ADAM12; COL6A1; PLCB1; MYL9; VCL; CCL13; CD274; SUCNR1; CALCRL; SEL1L; PDGFB; TGFA; CRHR2; BCL2L11; SOCS1; UBD; CCL4; CCL3; CKB; CCL17; P2RY12; SMAD1; LINGO1; PPP1R14A; CCL23; CCL22; HOMER2; FN1; LAMB1; INHBA; KTN1; SMAD7; ID3; PTPN7; RAMP1; PICALM
Interleukin-4 And Interleukin-13 Signaling R-HSA-6785807	9/107	6,90E-05	0,007598835	6	54	FCER2; CDKN1A; SOCS1; CCL22; MAOA; IRF4; ALOX15; FN1; F13A1
Metabolism Of Angiotensinogen To Angiotensins R-HSA-2022377	4/18	1,83E-04	0,017311945	17	149	MMP12; ACE; ANPEP; CTSZ
Adaptive Immune System R-HSA-1280218	26/733	2,12E-04	0,017557652	2	19	CD86; CD274; CD40; CD80; CLSTN1; CTSV; CD1C; CD1B; AP2A2; LILRA3; CD79A; SOCS1; MRC1; CTSC; AP2M1; CTSB; SIGLEC10; AP1B1; PDCD1LG2; CD200R1; CD209; TREML2; CD226; FCGR2B; HLA-DRB1; CLEC4G
Transport Of Small Molecules R-HSA-382551	25/706	2,88E-04	0,020334446	2	19	SLC22A4; SLC43A2; ABCB4; SEL1L; AQP6; SLC1A2; SLC3A2; SLC5A3; AP2A2; PCSK5; NIPAL1; LRRC8A; EMB; LRRC8B; CLIC2; AP2M1; SLC11A2; NIPA1; CTNS; TRPV1; SLC50A1;

						MCOLN2; SLC6A7; SLC7A8; SLC26A6
Chemokine Receptors Bind Chemokines R-HSA-380108	6/56	3,08E-04	0,020334446	7	59	CCL13; CCL22; CCL4; CCL3; CCR7; CCL17
Downregulated genes						
Neutrophil Degranulation R-HSA-6798695	13/468	5,30E-07	1,66E-04	6	93	FCN1; FPR1; CYBB; GGH; IQGAP2; MMP9; FCAR; CHIT1; BST1; ALOX5; PADI2; CHI3L1; LTA4H
Innate Immune System R-HSA-168249	17/1035	1,20E-05	1,87E-03	4	44	FCN1; C5AR2; FPR1; CYBB; GGH; IRAK3; IQGAP2; MMP9; FCAR; CHIT1; BST1; ALOX5; CASP1; PADI2; CHI3L1; LTA4H; CLEC4E
Immune System R-HSA-168256	23/1943	5,94E-05	6,20E-03	3	28	FCN1; COLEC12; IFITM2; C5AR2; FPR1; CYBB; GGH; IRAK3; IQGAP2; MMP9; FCAR; LMNB1; LILRA5; CHIT1; BST1; ALOX5; CASP1; PADI2; CHI3L1; PTK2B; SLAMF6; LTA4H; CLEC4E
Biosynthesis Of E-series 18(S)-resolvins R-HSA-9018896	2/5	2,40E-04	1,88E-02	137	1.140	ALOX5; LTA4H
Biosynthesis Of EPA-derived SPMs R-HSA-9018679	2/6	3,59E-04	2,25E-02	103	813	ALOX5; LTA4H
Biosynthesis Of DHA-derived SPMs R-HSA-9018677	2/17	0,00314275	0,153611871	27	158	ALOX5; LTA4H
Biosynthesis Of Specialized Proresolving Mediators (SPMs) R-HSA-9018678	2/19	0,00392618	0,153611871	24	134	ALOX5; LTA4H

Scavenging By Class A Receptors R-HSA-3000480	2/19	0,00392618	0,153611871	24	134	COLEC12; MARCO
Synthesis Of Leukotrienes (LT) And Eoxins (EX) R-HSA-2142691	2/21	0,0047907	0,166609931	22	115	ALOX5; LTA4H
Signaling By Interleukins R-HSA-449147	7/453	0,00727915	0,227837472	3	16	ALOX5; FPR1; CASP1; PTK2B; IRAK3; MMP9; LMNB1
